# Description of metabolic differences between castrated males and intact gilts obtained from high-throughput metabolomics of porcine plasma

**DOI:** 10.1093/jas/skaf178

**Published:** 2025-03-23

**Authors:** Samuele Bovo, Matteo Bolner, Giuseppina Schiavo, Giuliano Galimberti, Francesca Bertolini, Stefania Dall’Olio, Anisa Ribani, Paolo Zambonelli, Maurizio Gallo, Luca Fontanesi

**Affiliations:** Animal and Food Genomics Group, Division of Animal Sciences, Department of Agricultural and Food Sciences, University of Bologna, Bologna, Italy; Animal and Food Genomics Group, Division of Animal Sciences, Department of Agricultural and Food Sciences, University of Bologna, Bologna, Italy; Animal and Food Genomics Group, Division of Animal Sciences, Department of Agricultural and Food Sciences, University of Bologna, Bologna, Italy; Department of Statistical Sciences “Paolo Fortunati”, University of Bologna, Bologna, Italy; Animal and Food Genomics Group, Division of Animal Sciences, Department of Agricultural and Food Sciences, University of Bologna, Bologna, Italy; Animal and Food Genomics Group, Division of Animal Sciences, Department of Agricultural and Food Sciences, University of Bologna, Bologna, Italy; Animal and Food Genomics Group, Division of Animal Sciences, Department of Agricultural and Food Sciences, University of Bologna, Bologna, Italy; Animal and Food Genomics Group, Division of Animal Sciences, Department of Agricultural and Food Sciences, University of Bologna, Bologna, Italy; Associazione Nazionale Allevatori Suini, Roma, Italy; Animal and Food Genomics Group, Division of Animal Sciences, Department of Agricultural and Food Sciences, University of Bologna, Bologna, Italy

**Keywords:** castration, machine learning, metabolite, pig, plasma, *Sus scrofa*

## Abstract

Surgically castrated male pigs, which are commonly produced in pork production systems, have slightly lower production efficiency, compared to intact female pigs (gilts). This is mainly due to an unfavorable feed conversion rate and fatter carcasses. These differences influenced by physiological and genetic factors can be identified through metabolomics, which describes metabolic profiles. In this study, we used untargeted metabolomics to analyze the plasma of 694 Italian Large White pigs (228 castrated males and 466 intact gilts), sampled at slaughter. The metabolomic profiles included 731 metabolites covering 98 sub-pathways. The raw metabolomic data were cleaned and imputed using multivariate imputation by chained equations. The Boruta algorithm was then employed to identify metabolites that have different concentrations between castrated males and intact gilts. To address the random nature of feature selection, multiple Boruta runs were generated, and nested within a 10-fold cross-validation, resulting in 1,250 Boruta datasets. These datasets helped identify 40 informative metabolites, with a reduced core of 15 metabolites consistently confirmed across all runs. Their calculated random forest out-of-bag error was 0.25 and 0.27, respectively. The relevance, ranking, and predictive ability of each selected metabolite were determined based on the mean decrease Gini (**MDG**) and the area under the curve (**AUC**) of the receiver operating characteristic curve analysis, with MDG values of 0.024 ± 0.007 and 0.030 ± 0.009 and AUC values of 0.62 ± 0.04 and 0.65 ± 0.03 for the 2 metabolite sets, respectively. Of the 40 selected metabolites, 60% had higher concentrations in castrated males than in intact gilts, while in the 15 metabolites set, this percentage was 80%. Network and biological pathways analyses indicated that the selected metabolites were primarily amino acids and lipids, many of which belonged to their respective sub-pathways, suggesting minimal biological differences between castrated males and intact gilts. These findings support previous results obtained using a targeted metabolomic platform. This study represents the largest investigation to date on the pig sex metabolome, providing essential biological insights that could inform precise husbandry and feeding strategies in pigs, taking into consideration the castration status of the males.

## Teaser

Untargeted metabolomics, coupled with a specifically designed and robust statistical approach, described the plasma metabolome in a Large White pig population. This identified 40 plasma metabolites that discriminate between castrated males and intact gilts, opening novel avenues to describe physiological differences between these 2 groups of pigs.

## Graphical Abstract

**Graphical Abstract:**
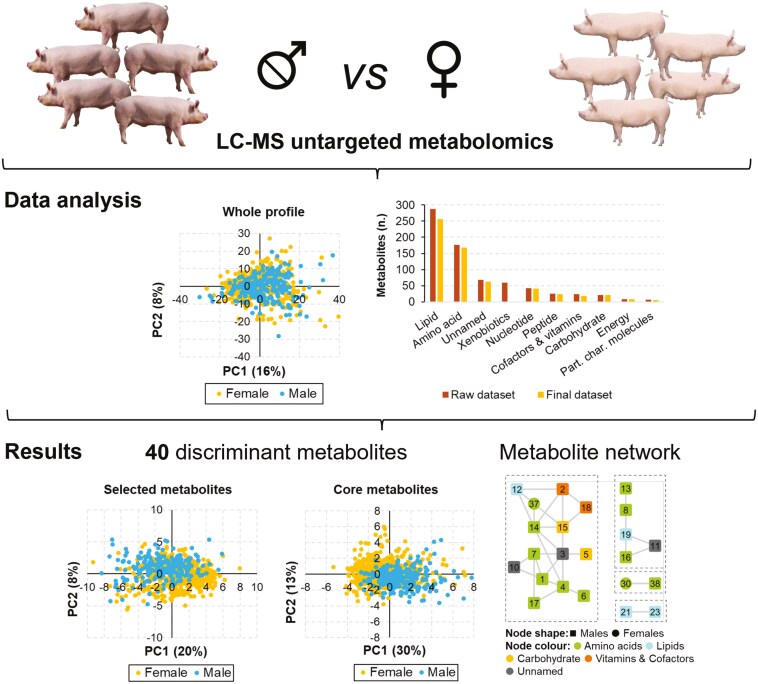

## Introduction

Sex is one of the most important factors that affects many aspects of pig production systems, including husbandry practices. It has significant impacts on all economically relevant traits and also raises ethical issues related to the castration of males (Bereskin and Davey, 1976; Dunshea, 2001; Latorre et al., 2003a, b, 2008; Trefan et al., 2013; Patience et al., 2015; Van Der Heide et al., 2016; Borell et al., 2020; Elbert et al., 2020; Squires et al., 2020). In this regard, normal male sexual development is closely linked to the well-known issue of boar taint in meat. As a result, male pigs are often surgically castrated in many production chains to prevent boar taint and ensure that the resulting pig meat is then marketable (De Briyne et al., 2016; CASTRUM consortium, 2017; Mateos et al., 2024). Surgically castrated males, however, have slightly lower production efficiency due to an unfavorable feed conversion rate and fatter carcasses compared to intact gilts. This has been shown in many studies across different breeds and production systems (D’Souza and Mullan, 2002; Gispert et al., 2010; Morales et al., 2011; Garitano et al., 2013; Puls et al., 2017). The production differences between surgically castrated males and intact gilts are relevant for several practical aspects, ranging from feeding to carcass grading. These differences may prevent the complete standardization of husbandry practices and can lead to inhomogeneity in slaughtered animals. Despite the importance of these effects, only a few studies have indirectly attempted to describe the biological mechanisms that determine these differences using molecular phenotypes (Orengo et al., 2014; Cai et al., 2015; Suárez-Belloch et al., 2015; Ohkoda et al., 2021; Moeser et al., 2022; Fardisi et al., 2023).

Metabolomics has emerged as a powerful tool for describing animal metabolism under specific conditions. By exploring a large set of small molecules circulating in a biofluid or present in tissue, metabolomics has proven to be effective in identifying novel molecular descriptors for dissecting the biological complexity of the phenomena under investigation in different species, including livestock (Fontanesi, 2016; Goldansaz et al., 2017; Hao et al., 2021; Imaz et al., 2022; Li et al., 2022). In pigs, metabolomics has been applied for various purposes, ranging from the general characterization of biofluids to more specific investigations. These studies include breed characterization, evaluation of feeding strategies on metabolism, description of the effects of stress conditions, and analysis of the effects of genetic variability on metabolite levels, among other studies (e.g., Cai et al., 2015; Bovo et al., 2016, 2023, 2025a, 2025b; Picone et al., 2018; Luise et al., 2020; Peukert et al., 2021; Wang et al., 2021; Song et al., 2022; Dervishi et al., 2023; Metzler-Zebeli et al., 2023). In a previous investigation, we initially explored metabolomic differences between castrated males and intact gilts from a performance-tested heavy pig population of Italian Large White pigs (Bovo et al., 2015). This was done through a targeted metabolomics approach that quantified approximately 180 plasma metabolites in finished 9-m-old pigs. Another study compared the serum metabolomic profiles of castrated males and intact females during the growing phase (Dervishi et al., 2021). The cited study analyzed approximately 50 metabolites using nuclear magnetic resonance. Recently, Peukert et al. (2021) reported metabolomic differences between intact males and gilts of crossbred pigs analyzed at the blood, muscle, and liver levels using untargeted metabolomic platforms.

In this study, we significantly expanded the information on the metabolomic plasma profiles of Italian Large White pigs using a high-throughput untargeted metabolic platform to measure approximately 700 metabolites, allowing for a more detailed characterization of the metabolomic differences between castrated males and intact gilts. The results indicated that these 2 groups of pigs have similar metabolomic profiles at the end of their productive life, with a few peculiar differences that could be valuable in describing their metabolic fingerprinting.

## Material and Methods

### Animal ethics

All animals used in this study were kept in compliance with Italian and European legislation regarding pig husbandry. The procedures described were in accordance with Italian and European Union regulations for animal care and slaughter. The pigs were slaughtered in a commercial abattoir following standard procedures. The pigs were not raised or treated in any way for the purpose of this study, so no additional ethical statement is necessary.

### Animals and blood collection

The metabolomic study was conducted on 694 Italian Large White pigs consisting of 466 intact gilts and 228 castrated males, which were slaughtered over 23 different days. These pigs were part of the Italian sib-testing program for the Italian Large White pig population. The program involved triplets of pigs from the same litter, which included 2 intact females and 1 castrated male. Each pig was individually performance-tested at the Central Station of the National Pig Breeder Association. The testing period lasted from 30 to 45 d of age to approximately 9 mo of age, until they reached a final live weight of 155 ± 5 kg. Throughout the testing period, all pigs were fed the same growing and finishing diets and were handled in a consistent manner. At the conclusion of the testing period, the pigs underwent a fasting period of approximately 12 h, were then transported to a commercial abattoir, and were slaughtered after electrical stunning in the morning at around 0800h.

Blood was collected into 50 ml tubes containing ethylenediaminetetraacetic acid (**EDTA**) from the draining carotid artery immediately after jugulation and exsanguination. The tubes were inverted 8 to 10 times and then centrifuged within 2 h at 2,420 × *g* for 10 min at 4 °C. Plasma samples were then divided into several aliquots and stored at −80 °C till subsequent analyses (Bovo et al., 2015).

### Metabolomics of plasma samples

Plasma samples were analyzed at Metabolon, Inc. (Durham, NC, USA) using untargeted metabolomics (the HD4 metabolomics panel). A methanol extraction protocol was used to precipitate proteins, dissociate small molecules bound to proteins, and recover metabolites. The resulting extract underwent various ultra-performance liquid chromatography–tandem mass spectrometry (**UPLC-MS/MS**) steps, including reverse phase (**RP**)/UPLC-MS/MS with positive ion mode electrospray ionization (**ESI**), RP/UPLC-MS/MS with negative ion mode ESI and Hydrophilic Interaction Liquid Chromatography (**HILIC**)/UPLC-MS/MS with negative ion mode ESI. Three types of controls (**QC**) were analyzed alongside the experimental samples: 1) a pooled sample generated from a small portion of each experimental sample, 2) extracted water samples, and 3) a cocktail of standards spiked into every analyzed sample. Raw data was extracted, peaks were identified, and QC was processed using Metabolon’s hardware and software. For each metabolite, the raw peak areas were divided by the median raw peak area of the QC samples. Details regarding the instruments used and the sample and data processing procedures carried out by Metabolon are provided in Text S1. Metabolite annotations, including chemical names, database identifiers (HMDB and KEGG), and biological super-pathways and sub-pathways, were provided by Metabolon as part of their standard annotation procedure.

A total of 731 metabolites were identified, including 662 named and 69 unnamed metabolites belonging to different metabolite classes encompassing 7 metabolic super-pathways: lipids, amino acids, nucleotides, peptides, carbohydrates, cofactors and vitamins, energy, and partially characterized molecules (Table S1). Unnamed metabolites are those that have been detected and measured, but their chemical identity has not yet been elucidated. Metabolites from the xenobiotic class were not included in the subsequent analyses.

### Data cleaning and imputation

Data was quality-checked and filtered as outlined in a previous work (Bovo et al., 2015). Briefly, outliers were identified for each metabolite, removed and marked as missing data (NA, i.e. not available) if abundances deviated more than 5 times the interquartile range below or above the median for the metabolite under evaluation. A metabolite was then removed from the dataset if it had more than 25% of NAs. A sample was removed from the dataset if it had more than 30% of NAs across the entire metabolomic profile.

**Figure 1.**
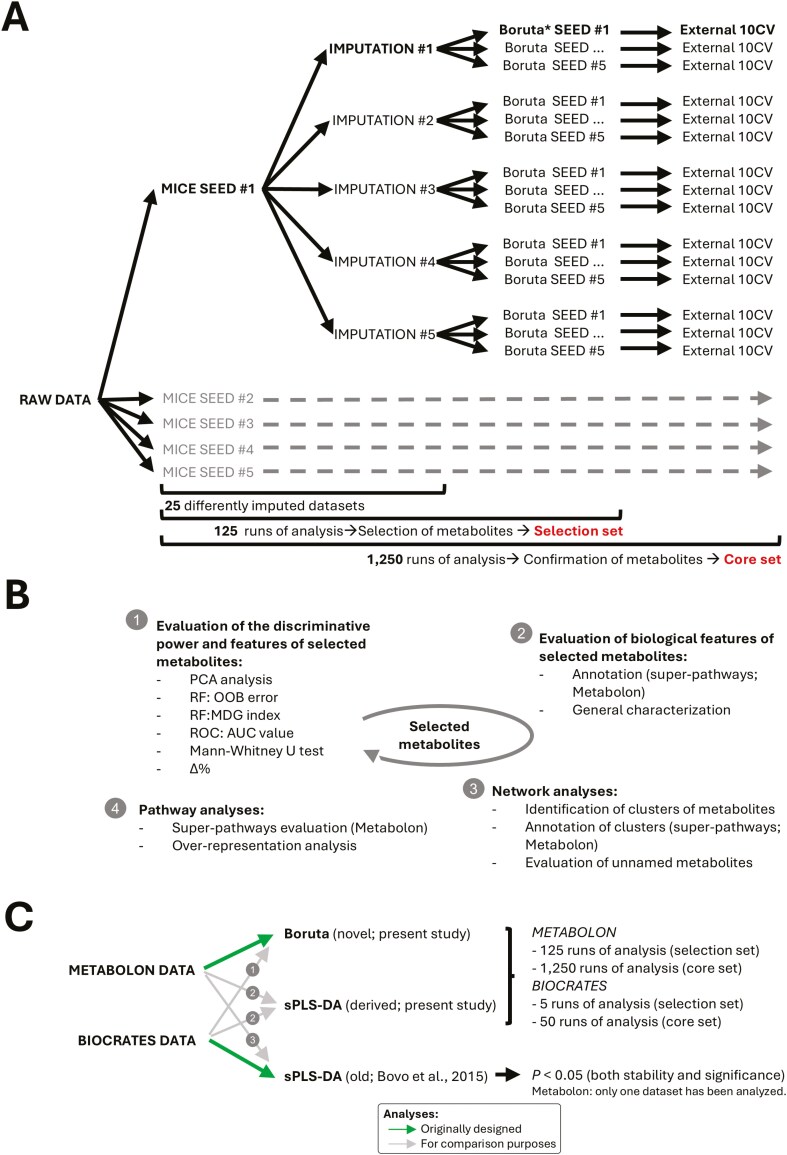
Metabolomic data analyses. A) Data analysis pipeline used for the identification of sex-influenced metabolites. It includes the use of 5 different random seeds in the imputation step (each generating 5 alternative datasets) for a total of 25 imputed datasets. Each dataset is then analyzed with Boruta with 5 different random seeds, for a total of 125 runs. Metabolites commonly selected across the 125 runs are retained representing the “selection set”. Each run is subsequently subjected to a 10CV procedure, for a total of 1,250 Boruta runs. Metabolites commonly selected across the 1,250 runs are retained representing the stably selected metabolite set, here define as “core set”. B) Methodological and biological evaluation of selected metabolites. This evaluation is characterized by 4 steps that define discriminative power and statistics that characterize selected metabolites, their biological features and functions, relationships and biological pathways. C) Comparative analysis and pipeline evaluation applied across metabolomic platforms (Metabolon and Biocrates datasets). It includes 3 other blocks of analysis: 1) a novel pipeline based on Boruta applied to the Biocrates dataset, 2) a novel pipeline replacing Boruta with sPLS-DA applied to both Metabolon and Biocrates datasets, and 3) a previously applied pipeline (old) based on sPLS-DA used to analyze the Metabolon dataset. Abbreviations: AUC, area under the curve; CV, cross-validation, MDG, mean decrease Gini; MICE, multivariate imputation by chained equations approach; OOB: out-of-bag; PCA, principal component analysis; ROC, receiver operating characteristic; RF, random forest; sPLS-DA, sparse partial least squares discriminant analysis.

The procedure described by Faquih et al. (2020), based on the multivariate imputation by chained equations (**MICE**) approach (Azur et al., 2011), was adopted in our data analysis pipeline to impute NAs (Fig. 1A). For each metabolite presenting NAs (target variable), the 10 most highly correlated endogenous metabolites (based on Pearson’s correlation coefficient) were selected as predictor variables for imputation. The imputation was then performed using the predictive mean matching algorithm, which is the default and recommended univariate imputation method used by MICE (Azur et al., 2011). Five different imputed datasets were produced as a result (IMPUTATION 1 to 5; Fig. 1A). Additionally, to account for the random component of the imputation process, we expanded the procedure by using 5 different random state seeds (SEED 1 to 5; Fig. 1A) resulting in a total of 25 imputed datasets. Confounding factors were then removed by regressing each metabolite on covariates (fixed effect), that are the animal weight and sampling day, as previously described by Bovo et al. (2015). Briefly, the model was $y_{i}=\;\beta_{0}\;+\beta_{w}w_{i}+\overset{J-1}{\underset{j=1}{\sum }}\beta_{Cj}d_{ij}+\xi_{i}\;$, where *y*_*i*_ denotes the metabolite concentration for the *i*th animal, β_0_ denotes the intercept term, *w*_*i*_ indicates the weight of the *i*th animal, *d*_*i*1_, …, *d*_*i*(*J*−1)_ is a set of *J* = 23 dummy variables coding the blood collection date for the *i*th animal, β_*w*_ and β_*Cj*_ are the corresponding regression coefficients and ξ_*i*_ is the error term. Residuals were then obtained and used in data analysis. Analyses were conducted in the R v.4.2.3 (R Core Team, 2024) and Python v3.11.7 environments.

### Identification of sex-influenced metabolites in pigs

The Boruta algorithm (Kursa et al., 2010), which is a wrapper for the random forest (**RF**) classification algorithm, was used to identify sex-influenced metabolites. Boruta was implemented in Python v3.11.7, utilizing the BORUTA_py and scikit_learn (Pedregosa et al., 2011) packages with default parameters, except for the max_iter parameter, which was increased to 1,000 (default: 100), and the alpha parameter, which was set to a more stringent value of 0.01 (default: 0.05) to ensure more robust statistics. To address the imbalance in class sample size, the RF model was initiated with the “balanced” class weight option. This means that the weight of the input data was adjusted to be inversely proportional to class frequency. Only metabolites labeled as “confirmed” by Boruta were considered relevant for classification.

Each of the 25 imputed datasets underwent a Boruta analysis. Additionally, to address the random nature of feature selection, 5 different random seeds were used in the analysis (Boruta SEED 1 to 5; Fig. 1A), resulting in a total of 125 Boruta runs as part of the data analysis pipeline (Fig. 1A). The stability of the selection of discriminant metabolites and the potential impact of the dataset sample composition on the feature selection were assessed by incorporating a 10-fold cross-validation (**CV**) procedure into the data analysis pipeline. Briefly, from each of the 25 imputed datasets, the CV process iteratively cycled through all 10-folds: in each iteration, one-tenth of the samples was excluded, and 5 Boruta runs with different random seeds were applied to the remaining nine-tenths. This process resulted in 25 × 5 × 10 = 1,250 additional Boruta runs (External 10CV; Fig. 1A). Based on the produced outputs, we defined 1) a first reduced informative metabolite set as the 1 containing metabolites consistently confirmed across the 125 runs, and 2) a second reduced informative metabolite set, indicated as the core metabolite set, consisting only of metabolites that were confirmed across all 1,250 runs. The data were analyzed both before and after metabolite selection using principal component analysis (**PCA**) (Fig. 1B, step 1) in the R v.4.2.3 environment(R Core Team, 2024).

Following the study by Schiavo et al. (2024), the Boruta approach was combined with a standard RF analysis to determine the classification performance of reduced informative metabolite sets (Fig. 1B, step 1). This allowed for the estimation of the out-of-bag (**OOB**) score and error to evaluate the prediction performance of Boruta and assess the ability of selected metabolites to accurately assign each animal to its sex. This metric provides a computationally convenient approach to evaluate the RF without the need for a testing dataset or CV procedures (Huang and Deng, 2021). Then, to score the importance of metabolites and rank them accordingly, we calculated the mean decrease Gini (**MDG**) index for each metabolite. The RF analyses were conducted in R v.4.2.3 (R Core Team, 2024) using the package “randomForest”.

The predictive ability of each metabolite was further evaluated through receiver operating characteristic (**ROC**) curve analysis (Fig. 1B, step 1), where the area under the curve (**AUC**) value was obtained as a summary metric of the ROC curve. These analyses were performed in R v.4.2.3 (R Core Team, 2024) using the package pROC.

Differences in metabolite abundance between sexes were tested for each metabolite using a univariate approach via a Mann–Whitney *U* test (Fig. 1B, step 1), as implemented in R v.4.2.3 (R Core Team, 2024). Metabolites that showed a Bonferroni-corrected *P *< 0.05 were considered statistically significant.

The relative difference in metabolite concentration [Δ%, (Bovo et al., 2016); Fig. 1B, step 1] between sexes was also calculated and expressed as $\Delta {\%}_{i}=\frac{{\bar{x}}_{i}^{M}-{\bar{x}}_{i}^{F}}{{\bar{x}}_{i}^{M}}\times 100$, where ${\bar{x}}_{i}^{M}$ and ${\bar{x}}_{i}^{F}$ are the average metabolite abundance of the *i*th metabolite in castrated males and intact gilts, respectively.

### Functional analysis of sex-influenced metabolites

Annotations provided by Metabolon were used to initially evaluate the biological functions of selected metabolites (Fig. 1B, step 2). A network based on Pearson’s correlation coefficients (*r*) was then constructed to examine the relationships among the selected metabolites (Fig. 1B, step 3). Correlation coefficients were calculated at both the population level (including sex as a fixed effect in the regression model used for data cleaning) and for each sex separately. The strongest coefficient among the 3 was included in the network if |*r*| ≥ 0.5 (indicating a medium correlation). The network was visualized with Cytoscape 3.0.1 (Shannon, 2003), and basic statistics, such as node degree and betweenness centrality, were calculated. The network was also annotated with the biological information from Metabolon and metabolite features obtained during the selection process.

Biological pathways were further analyzed through over-representation analysis (Fig. 1B, step 4), using MetaboAnalyst v.6.0 (Pang et al., 2022). The RaMP-DB resource, which contains a comprehensive collection of 3694 metabolic and lipid pathways (metabolite sets) from different databases [i.e., Reactome, WikiPathways, and KEGG (Zhang, 2018)] was interrogated. Metabolite identifiers from the Human Metabolome Database (**HMDB**) were used as the input set. Metabolite sets were considered over-represented if they had a false discovery rate (**FDR**) corrected *P *< 0.05 and included at least 2 metabolites from the input set.

### Comparative analysis and pipeline evaluation across metabolomic platforms

Pigs included in this study have also been previously analyzed with the targeted metabolomic Biocrates AbsoluteIDQ p180 kit (Biocrates Life Sciences AG, Innsbruck, Austria), which provided semiquantitative measurements for approximately 180 metabolites from 7 biochemical families [amino acids, biogenic amines, hexoses, acylcarnitines, sphingomyelins, phosphatidylcholines and lysophosphatidylcholines; (Bovo et al., 2015)]. Using targeted metabolomics data produced with this platform, Bovo et al. (2015) identified metabolite sex-related differences by applying a sparse partial least square discriminant analysis (**sPLS-DA**) approach. There is a partial overlap in terms of metabolites between the Biocrates and Metabolon platforms: based on filtered data, 54 metabolites (40%) of the metabolites of the Biocrates platform are also in the Metabolon panels (Table S2). Correlation coefficients between abundances obtained with the 2 platforms were moderately high (*r* > 0.6) for 87% of these metabolites. Additionally, the output of the 2 platforms is expressed in different ways. Therefore, here we addressed the comparisons between the results of the 2 platforms in terms of general outputs based on different statistical approaches. To obtain a proper comparison between the previous results obtained with the Biocrates platform and the results obtained here using the Metabolon platform, we used the following data analysis pipelines and algorithms (Fig. 1C): 1) the previously obtained Biocrates data were analyzed with the approaches implemented in this study, 2) that merged the method implemented in the previous study with the novel methodology developed in this study, and 3) that applied the statistical approach used in the previous work with the data produced in the current study:

1) We applied the Boruta algorithm to the results obtained with the Biocrates platform. This dataset included 132 metabolites after the quality check and filtering steps that were regressed to obtain residuals (Bovo et al., 2015). Five Boruta runs (as 5 random seeds were tested) for each of the 10CV procedures were launched, for a total of 50 Boruta runs (Fig. 1C; point 1).
2) We applied to both the Metabolon and Biocrates metabolomics panels the statistical approach here described, by replacing the Boruta algorithm with the sPLS-DA algorithm. However, as the sPLS-DA algorithm reports a regression coefficient (β) for each selected metabolite, we marked as confirmed only those metabolites that had an identical sign of β over the 125 runs. Similarly, the sign of β was also considered when evaluating the stability of the selection over the 1,250 runs (Fig. 1C; point 2).
3) We applied to the Metabolon panel our previously developed sPLS-DA statistical approach (Bovo et al., 2015), relying on a single run of sPLS-DA to select metabolite features, followed by permutation tests to evaluate their *P* at the stability and significance of the selection tests (Bovo et al., 2015) (Fig. 1C; point 3).

## Results

### Description of the plasma metabolomic profile in the Italian Large White population

**Figure 2.**
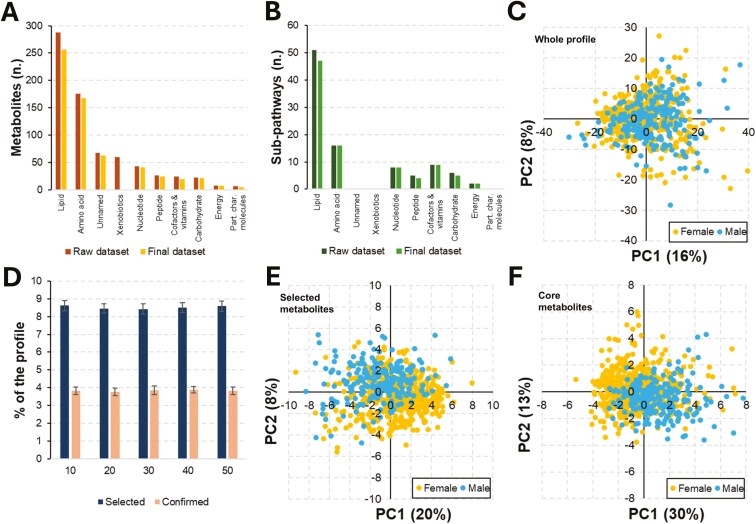
Metabolomics profile and sex-influenced metabolites. (A) Distribution of the analyzed metabolites across super-pathways; statistics are presented before and after data filtering. (B) Sub-pathways linked to the metabolomic profile as stratified by across super-pathways; statistics are presented before and after data filtering. (C) PCA before feature selection (No. 606 metabolites). (D) Statistics of selection (Boruta SEED 1 to 5); percentage relates to the entire filtered metabolite set (No. 606 metabolites). (E) PCA based on selected metabolites (No. 40). (F) PCA based on stably selected metabolites (core metabolite set; No. 15). Abbreviations: Part. char. molecules, partially characterized molecules; PCA, principal component analysis; PC, principal component.

Using the Metabolon platform, we obtained as raw data the relative quantification and identification of 731 metabolites: 1) 602 molecules (82%) were of endogenous origin, 2) 60 molecules (8%) were of xenobiotic origin, and 3) 69 molecules (9%), referred to as unnamed metabolites, had unknown origins. Endogenous metabolites encompassed various metabolisms, including 8 super-pathways (Metabolon): lipids (49% of the endogenous set), amino acids (29%), nucleotides (7%), peptides (4%), carbohydrates (4%), cofactors and vitamins (4%), energy (1%), and partially characterized molecules (1%). In total, the metabolites covered 98 sub-pathways (Metabolon; Table S1). Fig. 2A and B shows the distributions of metabolites across super-pathways and sub-pathways. Metabolites of xenobiotic origin were excluded from further analyses.

After the data cleaning procedures, 65 metabolites were discarded due to having more than 25% of missing data, while all samples were retained. This did not affect the proportions among the different super-pathways and sub-pathways (Table S1). The final dataset used for subsequent analyses included all animals (No. 694) and 606 metabolites covering the 8 super-pathways indicated above.

### Identification of metabolites differentiating castrated males and intact gilts

A PCA was initially conducted on the final metabolomic dataset to identify differences between the 2 sexes. The first 2 principal components (**PC**) explained 24% of the total variance (PC1 = 16% and PC2 = 8%), with no distinct clusters emerging; the clouds constituted by the pigs of the 2 sexes completely overlapped (Fig. 2C).

To identify sex-influenced metabolites, a data analysis pipeline was utilized, which first involved 125 runs of the Boruta algorithm to account for the influence of random state seed during data imputation and feature selection (5 random state seeds were used in the data imputation phase × 5 imputed datasets by MICE × 5 random state seeds were tested with Boruta). In total, 57 metabolites were selected (ranging from 47 to 56 in individual runs, with an average of 51; Fig. 2D), but only 40 metabolites (Table 1) were consistently identified in all 125 runs. To assess the stability of selection, an external 10-fold CV procedure was performed, increasing the number of Boruta runs to 1,250. Out of these, 15 metabolites (ranging from 21 to 26 metabolites in the individual runs, with an average of 23; Fig. 2D) were consistently selected across all 1,250 runs, forming a core set of metabolites indicative of differences between castrated males and intact gilts. The remaining 25 metabolites were confirmed in 58.5% to 99.8% of the 1,250 runs (with an average confirmation rate of 80.2 ± 14.7). These results indicated that while feature selection was stable, feature confirmation was less consistent when evaluating this specific condition (namely castrated males versus intact gilts). Among the 40 selected metabolites, 60% (24/40) had higher concentrations in castrated males while in the core metabolite set, this percentage increased to 80% (12/15).

**Table 1.** Sex-influenced metabolites (No. 40) identified by Boruta. Metabolites are sorted based on the AUC value (from the highest to the lowest)

General information about metabolites					Detection of metabolites						Network analysis		
Metabolite rank^1^	HMDB^2^	Metabolite name	Super pathway^3^	Sub-pathway^4^	Core set^5^	Sex^6^	Δ%^7^	MDG^7^	AUC^8^	*P-*value^9^	ASP^10^	BC^11^	ND^12^
1	0000679	Homocitrulline	AA	Urea cycle; Arginine and Proline Metabolism	Yes	M	27.7	0.060	0.70	3.7E−18	2.00	0.092	3
2	0003290	Gulonate	C&V	Ascorbate and Aldarate Metabolism	Yes	M	17.2	0.036	0.69	6.7E−16	2.23	0.035	4
3	–	X-23593	Unk	–	Yes	M	13.4	0.032	0.68	2.3E−14	1.54	0.394	6
4	–	1-Methyl-5-imidazolelactate	AA	Histidine Metabolism	Yes	M	16.7	0.031	0.67	1.2E−13	1.69	0.278	5
5	0240347	N6-carboxymethyllysine	Car	Advanced Glycation End-product	Yes	M	17.0	0.029	0.67	7.8E−13	2.46	0.000	1
6	04988	1-Methyl-5-imidazoleacetate	AA	Histidine Metabolism	Yes	M	34.4	0.032	0.66	2.1E−12	2.62	0.000	1
7	0003357	N-delta-acetylornithine	AA	Urea cycle; Arginine and Proline Metabolism	Yes	M	17.9	0.022	0.66	2.7E−12	2.08	0.084	4
8	0000294	Urea	AA	Urea cycle; Arginine and Proline Metabolism	Yes	M	13.0	0.028	0.66	2.7E−12	1.50	0.500	2
9	0002331	1-Ribosyl-imidazoleacetate	AA	Histidine Metabolism	Yes	M	20.5	0.018	0.65	4.3E−11	–	–	–
10	–	X-24736	Unk	–	No	M	18.6	0.022	0.65	1.8E−10	2.77	0.000	2
11	–	X-15503	Unk	–	Yes	M	17.9	0.031	0.65	3.0E−10	1.75	0.000	2
12	0240316	Glycerophosphoglycerol	Lip	Glycerolipid Metabolism	Yes	M	13.2	0.027	0.64	7.6E−10	2.31	0.004	3
13	0000092	Dimethylglycine	AA	Glycine, Serine and Threonine Metabolism	No	M	9.8	0.017	0.64	2.9E−09	2.25	0.000	1
14	0000206	N6-acetyllysine	AA	Lysine Metabolism	No	M	7.9	0.021	0.64	4.1E−09	1.62	0.300	6
15	0001851, 0000568, 0002917	Arabitol/xylitol	Car	Pentose Metabolism	No	M	10.3	0.018	0.64	7.0E−09	1.85	0.172	5
16	0000715	Kynurenate	AA	Tryptophan Metabolism	No	M	15.5	0.023	0.63	1.7E−08	1.75	0.000	2
17	0000479	3-Methylhistidine	AA	Histidine Metabolism	No	M	10.4	0.020	0.63	2.5E−08	2.38	0.009	2
18	0240294	2-O-methylascorbic acid	C&V	Ascorbate and Aldarate Metabolism	No	M	8.9	0.019	0.63	2.9E−08	2.62	0.000	2
19	0059766	Picolinoylglycine	Lip	Fatty Acid Metabolism (Acyl Glycine)	No	M	16.0	0.019	0.63	3.9E−08	1.25	0.667	3
20	0000670	Homoarginine	AA	Urea cycle; Arginine and Proline Metabolism	Yes	F	−15.2	0.026	0.62	2.6E−07	–	–	–
21	–	Tridecenedioate (C13:1-DC)	Lip	Fatty Acid, Dicarboxylate	Yes	M	21.9	0.025	0.62	8.1E−07	1.00	0.000	1
22	0002024	4-Imidazoleacetate	AA	Histidine Metabolism	No	M	20.1	0.027	0.61	8.7E−07	–	–	–
23	0094656	2-Hydroxydecanoate	Lip	Fatty Acid, Monohydroxy	No	M	19.8	0.020	0.61	1.3E−06	1.00	0.000	1
24	0000709	Cysteinylglycine disulfide	AA	Glutathione Metabolism	No	M	10.7	0.022	0.61	1.3E−06	–	–	–
25	0000248	Thyroxine	AA	Tyrosine Metabolism	No	M	7.9	0.029	0.61	6.5E−06	–	–	–
26		Glycosyl-N-palmitoyl-sphingosine (d18:1/16:0)	Lip	Hexosylceramides (HCER)	No	F	−11.5	0.019	0.60	3.5E−05	–	–	–
27	00653, 0000653	Cholesterol sulfate	Lip	Sterol	No	F	−12.1	0.022	0.59	5.2E−05	–	–	–
28	–	6-Bromotryptophan	AA	Tryptophan Metabolism	No	F	−10.0	0.024	0.59	5.4E−05	–	–	–
29	0028930	Leucylhydroxyproline	Pept	Dipeptide Derivative	Yes	F	−28.5	0.028	0.59	8.4E−05	–	–	–
30	0000187	Serine	AA	Glycine, Serine and Threonine Metabolism	No	F	−4.2	0.022	0.59	1.9E−04	1.00	0.000	1
31	0002869	Campesterol	Lip	Sterol	No	F	−10.0	0.020	0.59	2.5E−04	–	–	–
32		X-23639	Unk	–	No	F	−9.1	0.018	0.59	2.7E−04	–	–	–
33	0000014	2’-Deoxycytidine	Nucl	Pyrimidine Metabolism, Cytidine containing	No	F	−6.2	0.019	0.58	2.9E−04	–	–	–
34	0002166	3-Aminoisobutyrate	Nucl	Pyrimidine Metabolism, Thymine containing	No	F	−9.8	0.022	0.58	5.4E−04	–	–	–
35	0001434	3-Methoxytyrosine	AA	Tyrosine Metabolism	No	F	−6.5	0.022	0.58	5.6E−04	–	–	–
36	0002064	N-Acetylputrescine	AA	Polyamine Metabolism	No	F	−10.0	0.019	0.57	1.7E−03	–	–	–
37	0004620	N-Acetylarginine	AA	Urea cycle; Arginine and Proline Metabolism	Yes	F	−10.9	0.020	0.57	1.9E−03	2.31	0.004	3
38	0000167	Threonine	AA	Glycine, Serine and Threonine Metabolism	No	M	−3.7	0.020	0.56	8.4E−03	1.00	0.000	1
39	0001344	4-Hydroxyglutamate	AA	Glutamate Metabolism	No	F	−5.2	0.023	0.54	5.5E−02	–	–	–
40	–	N-Acetyl-isoputreanine	AA	Polyamine Metabolism	No	F	−4.8	0.024	0.54	7.1E−02	–	–	–

^1^Metabolites are ranked by the AUC. This identifier is used also for identifying nodes in the network.

^2^Human Metabolome Database identifier.

^3^Super-pathway as defined by the Metabolon platform. AA: amino acid; Car: carbohydrate; C&V: cofactors and vitamins; Lip: lipid; Nucl: nucleotide; Pept: peptide; Unk: unknown.

^4^Sub-pathway as defined by the Metabolon platform.

^5^Metabolites that passed the 10CV procedure (selected 1,250 times) define the core metabolite set.

^6^Relative difference in concentration between sexes. A positive value indicates higher concentrations in castrated males. A negative value indicates higher concentrations in intact gilts. M: higher concentration in castrated males; F: higher concentration in intact gilts.

^7^Mean decrease Gini index in RF analysis.

^8^Area under the curve of the ROC analysis.

^9^ *P* at the Mann–Whitney *U* test.

^10^Average shortest path. This statistic is available only for connected nodes and not for singletons (defined by the symbol “–”).

^11^Betweenness centrality. This statistic is available only for connected nodes and not for singletons (defined by the symbol “–”).

^12^Node degree. This statistic is available only for connected nodes and not for singletons (defined by the symbol “–”).

The discriminative power of selected metabolites was evaluated using different indices: 1) the OOB score (number of correctly predicted data), which gives an overall estimation of the prediction error and 2) the MDG and AUC values, which provide relevance and power at the single metabolite level. Additionally, the results of a Mann–Whitney *U* test (univariate statistics) were also evaluated. When considering the set of 40 metabolites, the OOB score was found to be 0.75 (OOB error = 0.25). The MDG index was calculated to be 0.024 ± 0.007 (min = 0.016; max = 0.060), while the AUC was determined to be 0.62 ± 0.04 (min = 0.54; max = 0.70). Homocitrulline, an amino acid belonging to the urea cycle and arginine and proline metabolism, emerged as the metabolite with the highest importance and discriminating power (MDG = 0.06; AUC = 0.70).

The correlation between MDG and AUC values was moderate (*r *= 0.55). At the Mann–Whitney *U* test, 70% of metabolites (No. 28) significantly differed (Bonferroni-corrected *P <* 0.05; Table 1). A relationship between AUC and Mann–Whitney *U*-statistic (*P*) was also identified (Fig. S1), as previously shown by Mason & Graham (Mason and Graham, 2002). PCA based on this set of metabolites did not show 2 clearly separated clusters (Fig. 2E).

Core metabolites had similar but lower OOB scores, equal to 0.73 (OOB error = 0.27). The MDG index and AUC values had higher average values of 0.030 ± 0.009 and 0.65 ± 0.03, respectively. Only 2 core metabolites did not pass the Mann–Whitney *U* test. Overall, statistically significant differences (*P *< 0.05) in MDG and AUC values were observed between the 15 core metabolites and the remaining 25, indicating that stable metabolites were generally better for classification. PCAs based on the core metabolite set did not show 2 clearly separated clusters. However, the percentage of explained variance based on the first 2 PCs reached 43% when considering the core metabolite set (Fig. 2F).

### Biological properties of discriminant metabolites

**Figure 3.**
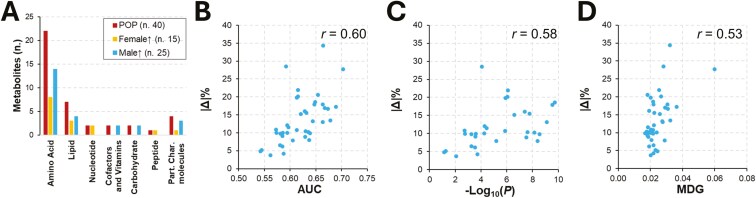
Biological features and direction of plasma concentration for the 40 selected metabolites. (A) Distribution of metabolites across super-pathways; data are presented for the whole population (POP; No. 40 metabolites) and stratified by sex [No. 15 metabolites with higher abundance in intact gilts (Female ↑) and the remaining No. 25 metabolites with higher abundance in castrated male (Male ↑)]. (B) Relationship between |Δ|% and AUC statistics. (C) Relationship between |Δ|% and Mann–Whitney *U*-statistic (*P*). (D) Relationship between |Δ|% and MDG statistics.

The 40 selected metabolites encompassed various super-pathways (Fig. 3A) and were distributed as follows: amino acids (No. 22) accounted for 55%, followed by lipids (No. 7; 17.5%), carbohydrates (No. 2; 5%), cofactors and vitamins (No. 2; 5%), nucleotides (No. 2; 5%), and peptides (No. 1; 2.5%). Unnamed molecules (No. 4) accounted for 10% and no metabolites from the energy super-pathway were selected. Core metabolites had a similar distribution across the same super-pathways (Fig. 3A).

When stratified by sex, 62% of the whole set of selected metabolites had higher concentrations in castrated males. More than half of amino acids (57%) and lipids (63%) had higher concentrations in castrated males. Cofactors and vitamins and carbohydrates were assigned to castrated males in terms of higher concentration, while nucleotides and peptides were assigned to intact gilts.

Based on the Δ% (Table 1), absolute values ranged from 3.7 (threonine) to 34.4 (1-methyl-5-imidazoleacetate), with an average value of 13.6 ± 6.7. When considering the |Δ|% values of core metabolites, the minimum was 10.9 (N-acetylarginine) and an average value of 19.0 ± 6.4 was obtained. We observed medium correlations between |Δ|% and AUC (*r *= 0.60; Fig. 3B), |Δ|% and the Mann–Whitney *U* -statistic (*r *= 0.58; Fig. 3C) and |Δ|% and MDG (*r *= 0.53; Fig. 3D). A total of 6 metabolites had a |Δ|% > 20. These metabolites were 1) more abundant in castrated males (5 out of 6) except for the dipeptide leucylhydroxyproline, 2) mainly included amino acids (4 out of 6), with most of them (3 out of 4; 4-imidazoleacetate, 1-ribosyl-imidazoleacetate and 1-methyl-5-imidazoleacetate) belonging to histidine metabolism. Except for 4-imidazoleacetate, all these metabolites were included in the core metabolite set.

### Functional characterization of discriminant metabolites

Based on Metabolon annotations, discriminant metabolites were mapped onto 26 sub-pathways (Metabolon). In particular, the 3 most populated ones (with at least 3 molecules each per sub-pathway) were 1) histidine metabolism (5 molecules), 2) urea cycle/arginine and proline metabolism (5 metabolites), and 3) glycine, serine and threonine metabolism (3 metabolites). Most of the other sub-pathways included 2 metabolites each. The 5 less populated sub-pathways were 1) polyamine metabolism, tryptophan metabolism, and tyrosine metabolism, which are all components of the amino acid super-pathway, 2) sterol metabolism, part of the lipids super-pathway, and 3) ascorbate and aldarate metabolism, within the cofactors and vitamins super-pathway. It is worth mentioning that all the metabolites of the urea cycle/arginine and proline metabolism and 3 out of 5 metabolites of the histidine metabolism, were listed within the core metabolite set.

Biological pathways were also studied through over-representation analysis. A total of 31 metabolites had a HMDB identifier and 29 of them were successfully mapped to the pathway libraries and metabolite sets available in MetaboAnalyst v.5.0. Only 2 functional sets showed an FDR-corrected *P <* 0.05. The most statistically significant set (*P =* 0.004) was related to biomarkers for urea cycle disorders (WikiPathways: WP4583), and annotated 4 metabolites from the input set, strengthening the characterization based on Metabolon information. However, it is worth noting that the annotation sets from Metabolon and WikiPathways did not completely overlap, with only urea and homocitrulline shared between the 2 annotation sets. The WikiPathways set also included L-threonine and 3-aminoisobutyrate. The second set (*P =* 0.0246) was the general set “biochemical pathways: part I” (WikiPathways: WP3604), annotating 9 of the selected metabolites.

### Metabolite correlation network

**Figure 4.**
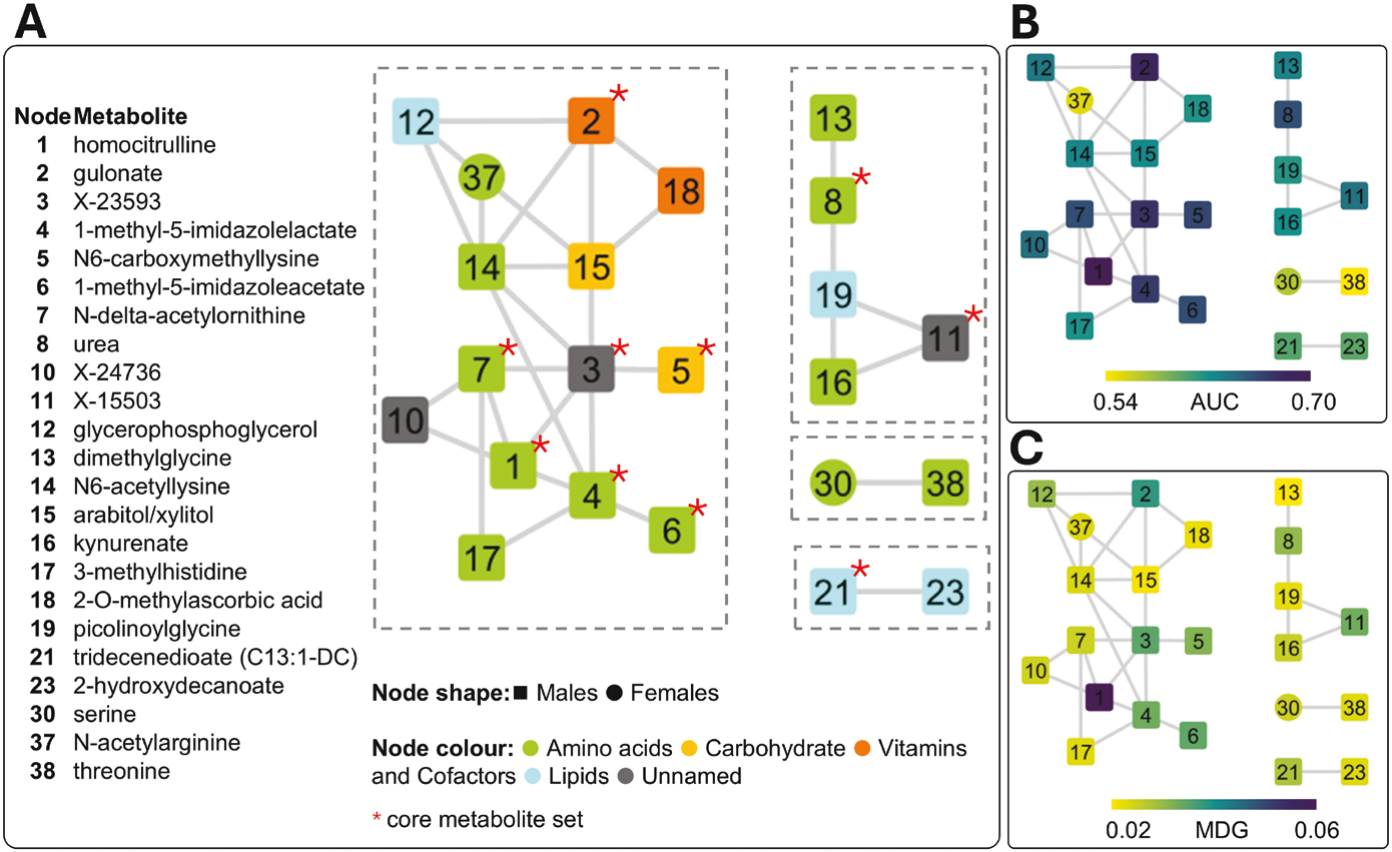
Correlation network obtained from selected metabolites (No. 40). Only connected components, involving No. 23 metabolites, are presented. Node identifiers are provided (details in Table 1). Shape of nodes refers to the metabolite abundance, higher either in castrated males (squared box) or in intact gilts (circle). Core metabolites are indicated with a star (*) symbol. (A) Metabolites are colored by super-pathway (annotations provided by Metabolon). (B) Metabolites are colored based on the AUC value from receiver operating characteristic analysis. (C) Metabolites are colored based on the MDG value.

Discriminant metabolites were studied in the context of their relationships through the generation of a correlation network, retaining only moderate to high correlations (|*r*| > 0.5) from 3 correlation sets (*r* at the entire population, castrated male, and intact gilts levels). In this network (Fig. 4A), more than half of the selected metabolites (23 out of 40) were connected to each other, totaling 31 edges. Four connected components emerged, including a large cluster of 14 metabolites, a medium cluster of 5 metabolites, and 2 small clusters, each containing 2 metabolites (Fig. 4A). Nine of these metabolites were included in the core metabolite set (Fig. 4A, red star symbol).

Metabolite clusters were evaluated in relation to biological and non-biological features. The large and medium clusters consisted of molecules from different super-pathways (Metabolon), highlighting several interclass links. These 2 clusters included most of the metabolites with the highest discriminative power (AUC and MDG; Fig. 4B and C, respectively) and the first 2 highest correlation coefficients. The strongest correlation at the population level (*r *= 0.79) was observed between kynurenate (node No. 16) and the unnamed metabolite X-15503 (node No. 11) within the medium size cluster. Studies in humans have associated these 2 metabolites with the kynurenine 3-monooxygenase (*KMO*) and kynureninase (*KYNU*) genes (Surendran et al., 2022; Chen et al., 2023), which encode 2 enzymes of the kynurenine pathway, functionally supporting the link between these 2 molecules. The second strongest correlation at the population level (*r *= 0.62) was observed between homocitrulline (node No. 1) and the unnamed metabolite X-23593 (node No. 3), within the larger cluster. A study in humans (Schlosser et al., 2020) associated the N-Acetyltransferase 8 (Putative) (*NAT8*) and ALMS1 Centrosome And Basal Body Associated Protein (*ALMS1*) genes with both metabolites, functionally linking them. Moreover, homocitrulline was also linked to the unnamed compound X-24736 (*r *= 0.58), and the same study, by Schlosser et al. (2020), associated both metabolites with the Solute Carrier Family 7 Member 9 (*SLC7A9*) gene. The third strongest correlation at the population level (*r *= 0.62) was observed between the unnamed metabolite X-23593 (node No. 3) and 1-methyl-5-imidazolelactate (node No. 4). The 2 small clusters were more specific to the metabolite class, with 1 related to lipids and the other to amino acids but containing metabolites with generally lower discriminative power.

By annotating the network (Fig. 4) with sub-pathway information (Metabolon), we observed the following connections: 1) 3 out 5 metabolites belonged to the histidine metabolites and were linked to each other (nodes Nos. 4, 6, 17), 2) 2 out of 4 metabolites belonged to the urea cycle / arginine and proline metabolism and were linked to each other (nodes Nos. 1 and 7), 3) 2 out of 3 metabolites belonged to the glycine, serine, and threonine metabolism and formed 1 of the 2 small clusters (nodes Nos. 30 and 38), and 4) 2 metabolites of the ascorbate and aldarate metabolism were directly connected (nodes Nos. 2 and 18).

When studying network properties, including the average shortest path, node degree, and centrality (Table 1), relevant no significant relationships between node features and metabolite importance (MDG), AUC, or |Δ|% were observed. However, it was noted that relevant metabolites tend to form links with each other (Fig. 4B and C).

### Comparative analyses across metabolomic platforms and data analysis pipelines

In our previous work (Bovo et al., 2015), 85 out of the 132 (64.4%) metabolites of the Biocrates panel were identified as sex-influenced by applying a data analysis pipeline relying on the sPLS-DA algorithm and considering 2 indices (i.e., the *P* of stability and the *P* of significance; Table S3) that were derived to evaluate the goodness of the selection. Considering the large difference between the Biocrates and Metabolon metabolomic panels, to obtain an integrated even general overview of sex-influenced metabolites, we carried out some additional tests (Fig. 1C). 1) We re-analyzed the Biocrates data with the novel pipeline (Boruta) developed here for the Metabolon data. 2) Because of previous analyses of this dataset relied on the sPLS-DA algorithm (Bovo et al., 2015), sPLS-DA was also integrated and tested in the current pipeline as a replacement of the Boruta algorithm and this approach was used to re-analyze both Biocrates and Metabolon datasets. 3) We finally run the previously used pipeline based on a simple sPLS-DA (Bovo et al., 2015) to re-analyze the Metabolon dataset.

**Figure 5.**
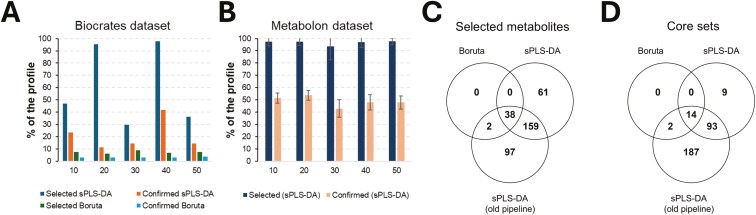
Comparative analyses across metabolomic platforms and data analysis pipelines. (A) Analysis of the Biocrates dataset. The novel developed pipeline tested both Boruta and sparse sPLS-DA as feature selection algorithms. (B) Analysis of the Metabolon dataset by using the developed pipeline with sPLS-DA as feature selection algorithm (Boruta SEED 1 to 5). (C) Analysis of the Metabolon dataset: overlap between selected metabolites coming from the different data analysis pipelines. (D) Analysis of the Metabolon dataset: overlap between selected metabolites populating the “core sets”.

1) In the analysis of the Biocrates panel (Table S3), the novel Boruta pipeline led to select a total of 14 metabolites (from 8 to 10 metabolites/seed; Fig. 5A), with 6 of them (C3, kynurenine, PC aa C40:6, PC ae C36:1, PC ae C36:5, and serotonin) shared among the 5 tested random seeds. Four metabolites (C3, PC aa C40:6, PC ae C36:5, and serotonin) were confirmed across the 50 Boruta runs that included the 10CV procedure (core set), all of them overlapping the 85 biomarkers initially discovered. As previously described, also in this case the results obtained highlighted how our pipeline, when relying on Boruta, stably selects the most relevant features and counteracts random effects (Fig. 5A). Considering the AUC (Table S3), values were in the ranges 0.54 to 0.62, 0.59 to 0.62, and 0.59 to 0.62 for the selected, shared and confirmed metabolite sets. 2) When Boruta was replaced with sPLS-DA (Table S3), selection resulted instable: from 39 to 129 metabolites/seed (Fig. 5A), for a total of 129 metabolites (>97%) selected at least by 1 seed and 39 in common to all the 5 seeds. However, only 34 of them had a stable direction of the β coefficient and 11 of these metabolites (His, Kynurenine, Met-SO, PC aa C32:2, PC ae C36:5, PC ae C44:6, SM (OH) C16:1, SM C18:1, Ser, Serotonin and Taurine), representing the core set, were confirmed across the 50 Boruta runs and overlapped the 85 biomarkers previously discovered (Bovo et al., 2015). In this case, AUC were in the ranges 0.46 to 0.62, 0.46 to 0.62, and 0.56 to 0.60 for the selected, shared and confirmed metabolite sets. Considering the 2 core sets, only 2 molecules (Serotonin and PC ae C36:5; AUC equal to 0.59 and 0.60, respectively) were common to both.

Considering the Metabolon panel, replacement of Boruta with sPLS-DA in the pipeline resulted in selecting almost the whole profile for each tested random seed and almost half of the profile was then confirmed for each random seed (Fig. 4B). A total of 374 metabolites were always selected across the 125 runs and 16 metabolites resulted lastly confirmed across all the 1,250 runs (with also a stable direction of the β coefficient), representing the core set. The AUC for these 2 sets were in the range 0.47 to 0.71 for both.

The application of our previously developed statistical approach to the Metabolon dataset led to obtaining 445 metabolites with *P* of stability < 0.05 and 337 metabolites with *P* of significance < 0.05. Considering both indices simultaneously, 296 metabolites could be marked as sex influenced. The AUC values for these 2 sets were in the range 0.47 to 0.71. Summarizing the results obtained with different statistics reported in the analysis of Metabolon dataset, wide differences emerged between the adopted algorithms and pipelines. In fact, the number of selected metabolites progressively increased passing from 40 (novel pipeline: based on Boruta 125 runs) to 374 (novel pipeline: based on sPLS-DA 125 runs and with stable β direction) and 445 (previously applied pipeline: sPLS-DA with both *P <* 0.05). Fig. 5C shows the overlap between the different selected sets and highlights that 1) almost all metabolites captured by Boruta are also captured by the sPLS-DA approaches and 2) the previous implementation of sPLS-DA captures more metabolites than the novel one. Similarly, we evaluated the core sets: we moved from 15 metabolites (novel pipeline; 1,250 runs of Boruta), to 16 (novel pipeline; 1,250 runs of sPLS-DA and stable β direction) and 296 (old pipeline, sPLS-DA with *P <* 0.05), with most of the metabolite captured by Boruta that are captured also by the sPLS-DA approaches (Fig. 5D).

## Discussion

In this study, we investigated sex-related differences in pig metabolism using a high-throughput untargeted plasma metabolomic approach. To date, only a few studies in pigs (Bovo et al., 2015; Dervishi et al., 2021; Peukert et al., 2021;  Ponsuksili et al., 2022) have evaluated sexual dimorphisms at the metabolome and metabolism levels. Our study has unique characteristics that set it apart from previous investigations. Firstly, we evaluated a large cohort of performance-tested animals from the Italian Large White breed, ensuring sufficient statistical power to detect differences between the 2 groups of pigs, despite a slight imbalance due to the number of intact gilts being roughly twice that of the castrated males. Secondly, our pig population consisted of either castrated males or intact gilts, allowing us to explore the sex-related architecture of metabolism resulting from a combination of 2 major components: 1) genetics, determined by the combinations of the sex chromosomes X and Y; 2) castration of males, which alters the biologically planned structure of the metabolism as determined by the chromosomal structures of the 2 groups of pigs (males: XY; and females: XX). Thirdly, although our study was initially focused on the common practice of male castration, the opportunity to evaluate these 2 groups of pigs enabled us to study the effects of hormone deficiency syndromes. This research has the potential to uncover new avenues in translational biomedical sciences and further establish the pig as a valuable model organism.

Castration of male pigs is commonly done to prevent boar taint in meat derived from sexually mature males (5 to 6 mo old) (De Briyne et al., 2016; CASTRUM consortium, 2017; Mateos et al., 2024). This practice lowers levels of 2 key hormones that cause boar taint: androsterone and skatole (Zamaratskaia and Squires, 2009). Male castration is crucial for the Italian pig production system, which specializes in producing heavy pigs that are slaughtered at approximately 170 kg of live weight when animals have reached at least 9 mo of age to produce protected designation of origin hams [such as Parma and San Daniele hams; (Bosi and Russo, 2004)]. However, despite the effect of male castration at the hormonal level (at least on skatole and androstenone) has been the matter of many studies, little is known about its global effect on molecular and metabolic architecture.

In addressing the identification of sex-influenced metabolites (which differentiate castrated males and intact gilts), we utilized Boruta, a RF wrapper. We chose this machine learning algorithm because it has been recognized as a solution for the *all-relevant problems* of feature selection, allowing for the identification of the full set of features that contain information usable for prediction, rather than just the minimal set of metabolites needed to create a predictive model (Kursa et al., 2010). In our previous study (Bovo et al., 2015), we obtained a preliminary analysis of sex-metabolome differences in the same pig population using a different metabolomic platform (Biocrates) and a different statistical approach (sPLS-DA). In the current study, we conducted several additional robust statistical tests to align the results from our previous study with the results obtained now with the Metabolon platform.

Following the evaluation of 606 metabolites on the Metabolon platform, encompassing 93 different metabolic pathways, we identified a total of 40 metabolites that differentiate between castrated males and intact gilts using Boruta. This set of metabolites proved to be highly reliable, as the data analysis pipeline we utilized was specifically designed to address the issue of randomness inherent in algorithms and procedures. The identified metabolites exhibited moderate to high discriminative power, with AUC values that correctly classified the sex of the pigs for 75% of the population (OOB score). However, these metabolites were not able to completely separate the 2 groups, as shown in the PCA analyses (Fig. 2E and F), even when a more robust set of 15 discriminative metabolites (the core metabolite set) was utilized. This outcome was somewhat expected and confirmed our previous findings (Bovo et al., 2015), which discussed the limitations of clusterization based on plasma metabolites detected by the Biocrates platform, compared to studies in humans (Mittelstrass et al., 2011). Despite some methodological differences, the separation between castrated males and intact gilts was poor compared to what was obtained in humans when (intact) sexually mature males were compared to females (Mittelstrass et al., 2011; Costanzo et al., 2022). Castration of male animals reduces the differences with females, although it does not completely eliminate all metabolic differences. This finding in castrated male pigs is also supported by a study conducted by De Siqueira et al. (2022) who evaluated the plasma metabolome of castrated men, through temporary pharmacologically induced castration via testosterone depletion. When considering non-sexually mature individuals, such as the infant human population (12 mo old) studied by Ellul et al. (2020), a certain degree of differentiation between males and females was observed. Similar results were also observed between intact male and female piglets (Peukert et al., 2021). Comparisons across species and studies are however difficult to align due to heterogeneity in the metabolomic platforms, experimental designs, and statistical approaches used for the final interpretation of the results. The inclusion of datasets from other pig breeds that adhere to the same design, utilize the same metabolomics platform, and employ a similar statistical approach could offer valuable insights into the metabolic characteristics that are inherently associated with sex differences, as well as those that may be unique to particular breeds or genetic backgrounds.

To overcome some of the limitations previously mentioned, we worked to compare the results obtained from our previous study (Bovo et al., 2015) that produced metabolomic profiles using the Biocrates-targeted metabolomic platform from the same pigs analyzed here. A total of 85 metabolites from the Biocrates platform emerged as being sex-differentiated. This number was reduced to 21 when more stringent *P* statistics were applied. When we used the novel pipeline based on sPLS-DA on the same Biocrates dataset, only 11 and 5 metabolites passed the selection, all of which were within the list of 85 or 21 metabolites, respectively. When Boruta was applied, only 6 or 2 metabolites were identified with the 5 seed-based analyses or only 4 metabolites were identified with the 10CV analyses, among the lists of the 85 and 21 metabolites, respectively. This highlighted a quite high instability in the identification of relevant features if the influence of randomness effects is not properly controlled (Fig. 5), as also previously observed by Bovo et al. (2025a). However, when the random nature of feature selection is addressed as we did in our Boruta and sPLS-DA pipelines, most of the metabolites detected with Boruta were also detected with sPLS-DA. This stability in feature selection was also reported when we used Boruta and sPLS-DA to analyze Metabolon dataset (Fig. 5C and D). We cannot exclude the possibility that part of this instability is due to additional factors, such as the long-term storage of samples (10 to 12 yr at −80 °C), which may promote degradation or alteration of metabolites (Wagner-Golbs et al., 2019). Another factor could be platform-specific analytical methods, including differences in detection sensitivity. Therefore, multiple factors could explain the differences observed in platform analyses, and isolating the specific contribution of storage time or platform is challenging. However, a correlation analysis of the abundance values of metabolites commonly identified by the 2 platforms showed that 87% of these metabolites had a strong correlation (*r* > 0.6). It is also important to note that while the Metabolon dataset was generated from a limited number of analytical batches, the Biocrates data were acquired across multiple plates and runs, potentially introducing higher inter-batch variability.

Using the Boruta approach implemented here on the Metabolon dataset, we identified 40 sex-influenced metabolites (whose plasma level was different between castrated males and intact gilts): 16 formed a core set of stable metabolites. This conservative approach makes it possible to detect only relevant and major metabolomic features that describe the metabolic differences between these 2 groups of pigs. More than half of the 40 sex-influenced metabolites are from the amino-acid super-pathway. Within this class, despite the Biocrates and Metabolon panels presenting only a partial overlap in terms of screened molecules (40% of Biocrates metabolites are also in the Metabolon panel), serine and threonine were identified as sex-influenced by both panels. These 2 metabolites are important components of the “Glycine, Serine, and Threonine Metabolism” and their relationship was also highlighted in the reconstructed metabolite network. However, they only showed moderate discriminant power between sexes in both types of datasets (as in the univariate test they did not present a strong significant difference). Other molecules from the amino-acid super-pathway, in particular the “Urea cycle; Arginine and Proline Metabolism” (e.g., homocitrulline and urea) were more important in the discrimination as they had medium-high AUC values (> 0.66). Moreover, this metabolism included a total of 5 metabolites (N-delta-acetylornithine, homoarginine, and N-acetylarginine, as selected metabolites molecules, and serine and threonine as reported earlier). Pathway enrichment analysis confirmed the importance of the urea metabolism in differentiating castrated males from intact gilts. It was also interesting to note that the mentioned amino acid pathways are closely linked as 1) threonine is shared by both pathways and 2) among the selected metabolites, dimethylglycine (a derivative of glycine and member of the glycine, serine, and threonine metabolism) was present. This result confirms what was reported by Bovo et al. (2015) who suggested that these 2 groups of pigs may differ in terms of amino acid metabolism and protein-construction-related functions, which in turn, may indicate differences in nitrogen uptake between castrated males and intact gilts. The reconstructed network made it possible to add 2 other unnamed molecules to these pathways: metabolites X-23593 and X-24736, that in the networks were linked to homocitrulline. In human blood, the levels of X-23593 and homocitrulline have been shown to be associated with variability in the *NAT8* and *ALMS1* genes whereas both X-24736 and homocitrulline levels have been associated with variability in the *SLC7A9* gene (Schlosser et al., 2020).

Among the amino acids-related metabolisms, the histidine metabolism emerged as the second most populated one in terms of number of metabolites. Most of the molecules of this metabolism were among those having the largest difference in terms of abundance between the 2 groups of animals, with a generally higher concentration in castrated males than intact gilts. Among the metabolites in this amino acid metabolism, 3-methylhistidine has been reported to have a higher concentration in human plasma and urine in rats due to the effect of castration in males (Jiao et al., 2009; De Siqueira Guedes et al., 2022).

The tryptophan metabolism was another amino acid-related metabolism that was affected: among their metabolites, the higher concentration of plasma kynurenate in pigs can be also paired with another unnamed metabolite (X-15503). This link may be derived from what has been already reported in humans where the combination of network analysis and genome-wide association analysis made it possible to assign X-15503 to the kynurenine pathway (Surendran et al., 2022; Chen et al., 2023). Considering that molecules belonging to the KP are involved in modulating the activity of the mammalian immune and central nervous systems (Savitz, 2020), this information can be used to better understand a potential effect of male castration on behavior and immunological differences between castrated males and intact gilts.

Castrated pigs have usually lower lean body mass compared to intact males, which means that they may use dietary proteins less efficiently for muscle growth (i.e., decreased rate of muscle protein synthesis) that could lead to an increase in amino acid oxidation and nitrogen waste as a result of an increased release of free amino acids from muscle tissue into the bloodstream (Cai et al., 2010; Ruiz-Ascacibar et al., 2019; Fernández-Fígares et al., 2023). The higher concentration in castrated males for several metabolites involved in the metabolism of various amino acids can be explained by this process.

Other interesting profiles can be obtained from the lipid super-pathways where fatty acids tended to have a higher concentration in castrated males whereas sterols tended to have a higher concentration in intact gilts. Castration in pigs is traditionally associated with increased fat deposition. This has been shown in many studies across different breeds and production systems (D’Souza and Mullan, 2002; Gispert et al., 2010; Morales et al., 2011; Garitano et al., 2013; Puls et al., 2017). However, it is important to distinguish between fat accumulation and circulating lipids, as they may behave differently due to differences in metabolic regulation and tissue-specific mechanisms. Among other metabolite families, in general nucleotides and peptides had higher concentrations in intact gilts whereas carbohydrates, cofactors, and vitamins had higher concentration in castrated males. Particularly, a few molecules of these latter classes (i.e., gulonate, 2-O-methylascorbic acid, and arabitol/xylitol) were found linked together in the reconstructed network confirming the biological links established between these molecules (see KEGG maps: 00040 and 01240): 1) gulonate is a precursor of ascorbate (vitamin C), explaining the link with 2-O-methylascorbic acid and 2) gulonate can be metabolized to arabitol/xylitol. Interestingly, studies in castrated rats and chickens have shown that the synthesis and distribution of ascorbic acid is under hormonal regulation (Dieter, 1969; Khandwekar et al., 1973). However, for a better understanding of metabolomic changes, analyzing the relevant tissues and integrating them with transcriptomic data would help reveal the regulatory networks and gene expression changes that drive these metabolisms.

This work establishes the groundwork for further investigations into the mechanisms that regulate differences at the sex level in pigs and other farm animals. Specifically, it is important to analyze the metabolomic profiles of entire and castrated male pigs to better understand the links between metabolic pathways and castration-induced physiological changes that cannot be fully understood by comparing castrated males with intact gilts. Additionally, despite some limitations, this study also creates new research opportunities, such as exploring the impact of these reported metabolomic differences on behavior and production, and how these findings can inform management and feeding strategies. In the context of adopting precision feeding strategies, split-sex feeding, which involves providing different diets to gilts and castrated males, can be used to better match nutrient supply with the physiological needs of pigs. This approach is justified by sex-related differences in feed intake behavior, growth performance, and feed conversion ratio. Moreover, it must be acknowledged that this practice can benefit pig production systems at 2 other levels: 1) it offers an opportunity to optimize economic returns (by maximizing growth efficiency and minimizing unnecessary nutrient oversupply), and 2) can reduce nitrogen excretion, thereby supporting more environmentally sustainable production systems. Thus, in this context, the metabolomic profiling of different sexes can provide valuable biological insights into the physiological responses to feeding of the animal, helping refine nutritional strategies. In turn, this information contributes to improving animal health, welfare, and production efficiency.

## Conclusions

This study represented the first evaluation at a large scale of sex-influenced plasma metabolomic features in pigs. We detected different compounds, mainly amino acids, differentially abundant between castrated males and intact gilts, providing a better understanding of the metabolic differences between these 2 groups of pigs. Metabolic differences may open novel avenues to better address nutritional needs of castrated males and intact gilts, which may result in the development of precise management practices and feeding strategies, contributing to the sustainability of pig production systems.

## Supplementary Data

Supplementary data are available at *Journal of Animal Science* online.

skaf178_suppl_Supplementary_Tables_S1-S3_Figure_S1

## Abbreviations

AUC: area under the curve
CV: cross-validation
EDTA: ethylenediaminetetraacetic acid
ESI: electrospray ionization
FDR: false discovery rate
HILIC: hydrophilic interaction liquid chromatography
HMDB: human metabolome database
KEGG: Kyoto encyclopedia of genes and genomes
MDG: mean decrease Gini
MICE: multivariate imputation by chained equations
NMR: nuclear magnetic resonance
OOB: out-of-bag
PC: principal component
PCA: principal component analysis
PDO: protected designation of origin
PMM: predictive mean matching
QC: quality control
ROC: receiver operating characteristic
RF: random forest
RP: reverse phase
sPLS-DA: sparse partial least squares discriminant analysis
UPLC-MS/MS: ultra-performance liquid chromatography–tandem mass spectrometry

## References

[CIT0001] Azur, M. J., E. A. Stuart, C. Frangakis, and P. J. Leaf. 2011. Multiple imputation by chained equations: what is it and how does it work? Int. J. Methods Psychiatr. Res. 20:40–49. doi: https://doi.org/10.1002/mpr.329 21499542 PMC3074241

[CIT0002] Bereskin, B., and R. J. Davey. 1976. Breed, line, sex and diet effects and interactions in swine carcass traits. J. Anim. Sci. 42:43–51. doi: https://doi.org/10.2527/jas1976.42143x

[CIT0003] Borell, E., M. Bonneau, M. Holinger, A. Prunier, V. Stefanski, S. Zöls, and U. Weiler. 2020. Welfare aspects of raising entire male pigs and immunocastrates. Animals. 10:2140. doi: https://doi.org/10.3390/ani10112140 33213105 PMC7698590

[CIT0004] Bosi, P., and V. Russo. 2004. The production of the heavy pig for high quality processed products. Ital. J. Anim. Sci. 3:309–321. doi: https://doi.org/10.4081/ijas.2004.309

[CIT0005] Bovo, S., G. Mazzoni, D. G. Calò, G. Galimberti, F. Fanelli, M. Mezzullo, G. Schiavo, E. Scotti, A. Manisi, A. B. Samoré, et al. 2015. Deconstructing the pig sex metabolome: targeted metabolomics in heavy pigs revealed sexual dimorphisms in plasma biomarkers and metabolic pathways. J. Anim. Sci. 93:5681–5693. doi: https://doi.org/10.2527/jas.2015-9528 26641177

[CIT0006] Bovo, S., G. Mazzoni, G. Galimberti, D. G. Calò, F. Fanelli, M. Mezzullo, G. Schiavo, A. Manisi, P. Trevisi, P. Bosi, et al. 2016. Metabolomics evidences plasma and serum biomarkers differentiating two heavy pig breeds. Animal. 10:1741–1748. doi: https://doi.org/10.1017/S1751731116000483 27055632

[CIT0007] Bovo, S., G. Schiavo, G. Galimberti, F. Fanelli, F. Bertolini, S. Dall’Olio, U. Pagotto, and L. Fontanesi. 2023. Comparative targeted metabolomic profiles of porcine plasma and serum. Animal. 17:101029. doi: https://doi.org/10.1016/j.animal.2023.101029 38064856

[CIT0008] Bovo, S., M. Bolner, G. Schiavo, G. Galimberti, F. Bertolini, S. Dall’Olio, A. Ribani, P. Zambonelli, M. Gallo, and L. Fontanesi. 2025a. High-throughput untargeted metabolomics reveals metabolites and metabolic pathways that differentiate two divergent pig breeds. Animal. 19:101393. doi: https://doi.org/10.1016/j.animal.2024.101393 39731811

[CIT0009] Bovo, S., A. Ribani, F. Fanelli, G. Galimberti, P. L. Martelli, P. Trevisi, F. Bertolini, M. Bolner, R. Casadio, S. Dall’Olio, et al. 2025b. Merging metabolomics and genomics provides a catalog of genetic factors that influence molecular phenotypes in pigs linking relevant metabolic pathways. Genet. Sel. Evol. 57:11. doi: https://doi.org/10.1186/s12711-025-00960-8 40050712 PMC11887101

[CIT0010] Cai, Z. W., X. F. Zhao, X. L. Jiang, Y. C. Yao, C. J. Zhao, N. Y. Xu, and C. X. Wu. 2010. Comparison of muscle amino acid and fatty acid composition of castrated and uncastrated male pigs at different slaughter ages. Ital. J. Anim. Sci. 9:e33. doi: https://doi.org/10.4081/ijas.2010.e33

[CIT0011] Cai, Z., L. Zhang, X. Jiang, Y. Sheng, and N. Xu. 2015. Differential miRNA expression profiles in the longissimus dorsi muscle between intact and castrated male pigs. Res. Vet. Sci. 99:99–104. doi: https://doi.org/10.1016/j.rvsc.2014.12.012 25591995

[CIT0012] CASTRUM consortium, Directorate-General for Health and Food Safety (European Commission). 2017. Pig castration: methods of anaesthesia and analgesia for all pigs and other alternatives for pigs used in traditional products. Publications Office of the European Union. doi: https://doi.org/10.2875/057159

[CIT0013] Chen, Y., T. Lu, U. Pettersson-Kymmer, I. D. Stewart, G. Butler-Laporte, T. Nakanishi, A. Cerani, K. Y. H. Liang, S. Yoshiji, J. D. S. Willett, et al. 2023. Genomic atlas of the plasma metabolome prioritizes metabolites implicated in human diseases. Nat. Genet. 55:44–53. doi: https://doi.org/10.1038/s41588-022-01270-1 36635386 PMC7614162

[CIT0014] Costanzo, M., M. Caterino, G. Sotgiu, M. Ruoppolo, F. Franconi, and I. Campesi. 2022. Sex differences in the human metabolome. Biol. Sex Differ. 13:30. doi: https://doi.org/10.1186/s13293-022-00440-4 35706042 PMC9199320

[CIT0020] D’Souza, D. N., and B. P. Mullan. 2002. The effect of genotype, sex and management strategy on the eating quality of pork. Meat Sci. 60:95–101. doi: https://doi.org/10.1016/s0309-1740(01)00112-7 22063110

[CIT0015] De Briyne, N., C. Berg, T. Blaha, and D. Temple. 2016. Pig castration: will the EU manage to ban pig castration by 2018? Porcine Health Manag. 2:29. doi: https://doi.org/10.1186/s40813-016-0046-x 28405455 PMC5382460

[CIT0018] Dervishi, E., I. Reimert, L. E. Van Der Zande, P. Mathur, E. F. Knol, and G. S. Plastow. 2021. Relationship between indirect genetic effects for growth, environmental enrichment, coping style and sex with the serum metabolome profile of pigs. Sci. Rep. 11:23377. doi: https://doi.org/10.1038/s41598-021-02814-x 34862433 PMC8642533

[CIT0017] Dervishi, E., X. Bai, M. K. Dyck, J. C. S. Harding, F. Fortin, J. C. M. Dekkers, and G. Plastow. 2023. GWAS and genetic and phenotypic correlations of plasma metabolites with complete blood count traits in healthy young pigs reveal implications for pig immune response. Front. Mol. Biosci. 10:1140375. doi: https://doi.org/10.3389/fmolb.2023.1140375 36968283 PMC10034349

[CIT0016] De Siqueira Guedes, J., I. Pla, K. B. Sahlin, G. Monnerat, R. Appelqvist, G. Marko-Varga, A. Giwercman, G. B. Domont, A. Sanchez, F. C. S. Nogueira, et al. 2022. Plasma metabolome study reveals metabolic changes induced by pharmacological castration and testosterone supplementation in healthy young men. Sci. Rep. 12:15931. doi: https://doi.org/10.1038/s41598-022-19494-w 36151245 PMC9508133

[CIT0019] Dieter, M. P. 1969. Hormonal control of the synthesis and distribution of ascorbic acid in cockerels (*Gallus domesticus*). Proc. Soc. Exp. Biol. Med. 130:210–213. doi: https://doi.org/10.3181/00379727-130-33523 5762501

[CIT0021] Dunshea, F. R. 2001. Sexual dimorphism in growth of sucking and growing pigs. Asian-Australas. J. Anim. Sci. 14:1610–1615. doi: https://doi.org/10.5713/ajas.2001.1610

[CIT0022] Elbert, K., N. Matthews, R. Wassmuth, and J. Tetens. 2020. Effects of sire line, birth weight and sex on growth performance and carcass traits of crossbred pigs under standardized environmental conditions. Arch. Anim. Breed. 63:367–376. doi: https://doi.org/10.5194/aab-63-367-2020 33178885 PMC7648295

[CIT0023] Ellul, S., A. -L. Ponsonby, J. B. Carlin, F. Collier, T. Mansell, P. Vuillermin, D. Burgner, and R. Saffery; Barwon Infant Study Investigator Team. 2020. Sex differences in infant blood metabolite profile in association with weight and adiposity measures. Pediatr. Res. 88:473–483. doi: https://doi.org/10.1038/s41390-020-0762-4 31952075

[CIT0024] Faquih, T., M. van Smeden, J. Luo, S. le Cessie, G. Kastenmüller, J. Krumsiek, R. Noordam, D. van Heemst, F. R. Rosendaal, A. van Hylckama Vlieg, et al. 2020. A workflow for missing values imputation of untargeted metabolomics data. Metabolites. 10:486. doi: https://doi.org/10.3390/metabo10120486 33256233 PMC7761057

[CIT0025] Fardisi, M., K. Thelen, A. Groenendal, M. Rajput, K. Sebastian, G. A. Contreras, and A. J. Moeser. 2023. Early weaning and biological sex shape long-term immune and metabolic responses in pigs. Sci. Rep. 13:15907. doi: https://doi.org/10.1038/s41598-023-42553-9 37741873 PMC10517948

[CIT0026] Fernández-Fígares, I., A. Haro, M. Lachica, L. Lara, I. Seiquer, and R. Nieto. 2023. Metabolic profile of growing immune-and surgically castrated Iberian pigs fed diets of different amino acid concentration. Animals. 13:2650. doi: https://doi.org/10.3390/ani13162650 37627441 PMC10451894

[CIT0027] Fontanesi, L. 2016. Metabolomics and livestock genomics: insights into a phenotyping frontier and its applications in animal breeding. Anim. Front. 6:73–79. doi: https://doi.org/10.2527/af.2016-0011

[CIT0028] Garitano, I., C. Liébana, E. F. de Vargas, A. Olivares, and A. Daza. 2013. Effect of gender on growth performance, carcass characteristics, meat and fat composition of pigs slaughtered at 125 kg of live weight destined to Teruel (Spain) ham production. Ital. J. Anim. Sci. 12:e16. doi: https://doi.org/10.4081/ijas.2013.e16

[CIT0029] Gispert, M., M. Àngels Oliver, A. Velarde, P. Suarez, J. Pérez, and M. Font I Furnols. 2010. Carcass and meat quality characteristics of immunocastrated male, surgically castrated male, entire male and female pigs. Meat Sci. 85:664–670. doi: https://doi.org/10.1016/j.meatsci.2010.03.021 20416805

[CIT0030] Goldansaz, S. A., A. Guo, T. Sajed, M. Steele, G. Plastow, and D. Wishart. 2017. Livestock metabolomics and the livestock metabolome: a systematic review. PLoS One. 12:null. doi: https://doi.org/10.1371/journal.pone.0177675 PMC5439675 28531195

[CIT0031] Hao, D., J. Bai, J. Du, X. Wu, B. Thomsen, H. Gao, G. Su, and X. Wang. 2021. Overview of metabolomic analysis and the integration with multi-omics for economic traits in cattle. Metabolites. 11:753. doi: https://doi.org/10.3390/metabo11110753 34822411 PMC8621036

[CIT0032] Huang, S., Deng, H., 2021. Data analytics: a small data approach. 1st ed. Boca Raton: Chapman and Hall/CRC; doi: https://doi.org/10.1201/9781003102656

[CIT0033] Imaz, J. A., S. García, and L. A. González. 2022. The metabolomics profile of growth rate in grazing beef cattle. Sci. Rep. 12:2554. doi: https://doi.org/10.1038/s41598-022-06592-y 35169253 PMC8847617

[CIT0034] Jiao, Q., A. M. Pruznak, D. Huber, T. C. Vary, and C. H. Lang. 2009. Castration differentially alters basal and leucine-stimulated tissue protein synthesis in skeletal muscle and adipose tissue. Am. J. Physiol. Endocrinol. Metab. 297:E1222–E1232. doi: https://doi.org/10.1152/ajpendo.00473.2009 19755668 PMC2781348

[CIT0035] Khandwekar, P. V., M. K. Deshpande, N. Nath, and M. C. Nath. 1973. Ascorbic acid metabolism in castrated rats. Metabolism. 22:1485–1489. doi: https://doi.org/10.1016/0026-0495(73)90016-4 4762625

[CIT0036] Kursa, M. B., A. Jankowski, and W. R. Rudnicki. 2010. Boruta – A system for feature selection. Fundam. Inform. 101:271–285. doi: https://doi.org/10.3233/fi-2010-288

[CIT0038] Latorre, M. A., P. Medel, A. Fuentetaja, R. Lázaro, and G. G. Mateos. 2003a. Effect of gender, terminal sire line and age at slaughter on performance, carcass characteristics and meat quality of heavy pigs. Anim. Sci. 77:33–45. doi: https://doi.org/10.1017/s1357729800053625

[CIT0039] Latorre, M. A., M. Nieto, and G. G. Mateos. 2003b. Effect of sex and terminal sire genotype on performance, carcass characteristics, and meat quality of pigs slaughtered at 117 kg body weight. Meat Sci. 65:1369–1377. doi: https://doi.org/10.1016/S0309-1740(03)00059-7 22063781

[CIT0037] Latorre, M. A., E. García-Belenguer, and L. Ariño. 2008. The effects of sex and slaughter weight on growth performance and carcass traits of pigs intended for dry-cured ham from Teruel (Spain). J. Anim. Sci. 86:1933–1942. doi: https://doi.org/10.2527/jas.2007-0764 18441085

[CIT0040] Li, J., Y. Wang, R. Mukiibi, B. Karisa, G. S. Plastow, and C. Li. 2022. Integrative analyses of genomic and metabolomic data reveal genetic mechanisms associated with carcass merit traits in beef cattle. Sci. Rep. 12:3389. doi: https://doi.org/10.1038/s41598-022-06567-z 35232965 PMC8888742

[CIT0041] Luise, D., S. Bovo, P. Bosi, F. Fanelli, U. Pagotto, G. Galimberti, G. Mazzoni, S. Dall’Olio, and L. Fontanesi. 2020. Targeted metabolomic profiles of piglet plasma reveal physiological changes over the suckling period. Livest. Sci. 231:103890. doi: https://doi.org/10.1016/j.livsci.2019.103890

[CIT0042] Mason, S. J., and N. E. Graham. 2002. Areas beneath the relative operating characteristics (ROC) and relative operating levels (ROL) curves: statistical significance and interpretation. Q. J. R. Meteorol. Soc. 128:2145–2166. doi: https://doi.org/10.1256/003590002320603584

[CIT0043] Mateos, G. G., N. L. Corrales, G. Talegón, and L. Aguirre. 2024. Invited review — pig meat production in the European Union-27: current status, challenges, and future trends. Anim. Biosci. 37:755–774. doi: https://doi.org/10.5713/ab.23.0496 38606453 PMC11016692

[CIT0044] Metzler-Zebeli, B. U., F. Lerch, F. Yosi, J. C. Vötterl, S. Koger, M. Aigensberger, P. M. Rennhofer, F. Berthiller, and H. E. Schwartz-Zimmermann. 2023. Creep feeding and weaning influence the postnatal evolution of the plasma metabolome in neonatal piglets. Metabolites. 13:214. doi: https://doi.org/10.3390/metabo13020214 36837833 PMC9960666

[CIT0045] Mittelstrass, K., J. S. Ried, Z. Yu, J. Krumsiek, C. Gieger, C. Prehn, W. Roemisch-Margl, A. Polonikov, A. Peters, F. J. Theis, et al. 2011. Discovery of sexual dimorphisms in metabolic and genetic biomarkers. PLoS Genet. 7:e1002215. doi: https://doi.org/10.1371/journal.pgen.1002215 21852955 PMC3154959

[CIT0046] Moeser, A. J., A. Roney, M. Fardisi, and K. Thelen. 2022. Biological sex: an understudied factor driving disease susceptibility in pigs. J. Anim. Sci. 100:skac146. doi: https://doi.org/10.1093/jas/skac146 35708590 PMC9202566

[CIT0047] Morales, J. I., L. Cámara, J. D. Berrocoso, J. P. López, G. G. Mateos, and M. P. Serrano. 2011. Influence of sex and castration on growth performance and carcass quality of crossbred pigs from 2 Large White sire lines. J. Anim. Sci. 89:3481–3489. doi: https://doi.org/10.2527/jas.2010-3357 21680787

[CIT0048] Ohkoda, T., K. Yoshida, D. Ijiri, and A. Ohtsuka. 2021. Effect of mixed rearing of barrows and gilts on backfat thickness and serum metabolite profiles of the Kagoshima‐Kurobuta (Berkshire) pig. Anim. Sci. J. 92:e13655. doi: https://doi.org/10.1111/asj.13655 34738692 PMC9285486

[CIT0049] Orengo, J., C. Villodre, J. Madrid, S. Martínez, M. J. López, M. D. Megías, L. Valera, and F. Hernández. 2014. Effect of dietary crude glycerin on growth performance, nutrient digestibility and hormone levels of Iberian crossbred pigs from 50 to 100kg body weight. Livest. Sci. 165:95–99. doi: https://doi.org/10.1016/j.livsci.2014.04.033

[CIT0050] Pang, Z., G. Zhou, J. Ewald, L. Chang, O. Hacariz, N. Basu, and J. Xia. 2022. Using MetaboAnalyst 5.0 for LC–HRMS spectra processing, multi-omics integration and covariate adjustment of global metabolomics data. Nat. Protoc. 17:1735–1761. doi: https://doi.org/10.1038/s41596-022-00710-w 35715522

[CIT0051] Patience, J. F., M. C. Rossoni-Serão, and N. A. Gutiérrez. 2015. A review of feed efficiency in swine: biology and application. J. Anim. Sci. Biotechnol. 6:33. doi: https://doi.org/10.1186/s40104-015-0031-2 26251721 PMC4527244

[CIT0052] Pedregosa, F., G. Varoquaux, A. Gramfort, V. Michel, B. Thirion, O. Grisel, M. Blondel, P. Prettenhofer, R. Weiss, V. Dubourg, et al. 2011. Scikit-learn: machine Learning in Python. J. Mach. Learn. Res. 12:2825–2830.

[CIT0053] Peukert, M., S. Zimmermann, B. Egert, C. H. Weinert, T. Schwarzmann, and D. A. Brüggemann. 2021. Sexual dimorphism of metabolite profiles in pigs depends on the genetic background. Metabolites. 11:261. doi: https://doi.org/10.3390/metabo11050261 33922306 PMC8146355

[CIT0054] Picone, G., M. Zappaterra, D. Luise, A. Trimigno, F. Capozzi, V. Motta, R. Davoli, L. Nanni Costa, P. Bosi, and P. Trevisi. 2018. Metabolomics characterization of colostrum in three sow breeds and its influences on piglets’ survival and litter growth rates. J. Anim. Sci. Biotechnol. 9:23. doi: https://doi.org/10.1186/s40104-018-0237-1 29527304 PMC5840723

[CIT0055] Ponsuksili, S., E. Murani, F. Hadlich, M. A. Iqbal, B. Fuchs, C. E. Galuska, A. Perdomo-Sabogal, F. Sarais, N. Trakooljul, H. Reyer, et al. 2022. Prenatal transcript levels and metabolomics analyses reveal metabolic changes associated with intrauterine growth restriction and sex. Open Biol. 12:220151. doi: https://doi.org/10.1098/rsob.220151 36102059 PMC9471991

[CIT0056] Puls, C. L., A. Rojo, P. D. Matzat, A. L. Schroeder, and M. Ellis. 2017. Behavior of immunologically castrated barrows in comparison to gilts, physically castrated barrows, and intact male pigs. J. Anim. Sci. 95:2345–2353. doi: https://doi.org/10.2527/jas.2016.1335 28727061

[CIT0057] R Core Team, 2024. R: a language and environment for statistical computing. R Foundation for Statistical Computing, Vienna, Austria.

[CIT0058] Ruiz-Ascacibar, I., P. Stoll, M. Kreuzer, and G. Bee. 2019. Dietary CP and amino acid restriction has a different impact on the dynamics of protein, amino acid and fat deposition in entire male, castrated and female pigs. Animal. 13:74–82. doi: https://doi.org/10.1017/S1751731118000770 29789036 PMC6316349

[CIT0059] Savitz, J. 2020. The kynurenine pathway: a finger in every pie. Mol. Psychiatry. 25:131–147. doi: https://doi.org/10.1038/s41380-019-0414-4 30980044 PMC6790159

[CIT0060] Schiavo, G., F. Bertolini, S. Bovo, G. Galimberti, M. Muñoz, R. Bozzi, M. Čandek‐Potokar, C. Óvilo, and L. Fontanesi. 2024. Identification of population‐informative markers from high‐density genotyping data through combined feature selection and machine learning algorithms: application to European autochthonous and cosmopolitan pig breeds. Anim. Genet. 55:193–205. doi: https://doi.org/10.1111/age.13396 38191264

[CIT0061] Schlosser, P., Y. Li, P. Sekula, J. Raffler, F. Grundner-Culemann, M. Pietzner, Y. Cheng, M. Wuttke, I. Steinbrenner, U. T. Schultheiss, et al; GCKD Investigators. 2020. Genetic studies of urinary metabolites illuminate mechanisms of detoxification and excretion in humans. Nat. Genet. 52:167–176. doi: https://doi.org/10.1038/s41588-019-0567-8 31959995 PMC7484970

[CIT0062] Shannon, P., A. Markiel, O. Ozier, N. S. Baliga, J. T. Wang, D. Ramage, N. Amin, B. Schwikowski, and T. Ideker. 2003. Cytoscape: a software environment for integrated models of biomolecular interaction networks. Genome Res. 13:2498–2504. doi: https://doi.org/10.1101/gr.1239303 14597658 PMC403769

[CIT0063] Song, B., C. Zheng, J. Zheng, S. Zhang, Y. Zhong, Q. Guo, F. Li, C. Long, K. Xu, Y. Duan, et al. 2022. Comparisons of carcass traits, meat quality, and serum metabolome between Shaziling and Yorkshire pigs. Anim. Nutr. 8:125–134. doi: https://doi.org/10.1016/j.aninu.2021.06.011 34977382 PMC8669263

[CIT0064] Squires, E. J., C. Bone, and J. Cameron. 2020. Pork production with entire males: directions for control of boar taint. Animals (Basel). 10:1665. doi: https://doi.org/10.3390/ani10091665 32947846 PMC7552340

[CIT0065] Suárez-Belloch, J., J. A. Guada, and M. A. Latorre. 2015. The effect of lysine restriction during grower period on productive performance, serum metabolites and fatness of heavy barrows and gilts. Livest. Sci. 171:36–43. doi: https://doi.org/10.1016/j.livsci.2014.11.006

[CIT0066] Surendran, P., I. D. Stewart, V. P. W. Au Yeung, M. Pietzner, J. Raffler, M. A. Wörheide, C. Li, R. F. Smith, L. B. L. Wittemans, L. Bomba, et al. 2022. Rare and common genetic determinants of metabolic individuality and their effects on human health. Nat. Med. 28:2321–2332. doi: https://doi.org/10.1038/s41591-022-02046-0 36357675 PMC9671801

[CIT0067] Trefan, L., A. Doeschl-Wilson, J. A. Rooke, C. Terlouw, and L. Bünger. 2013. Meta-analysis of effects of gender in combination with carcass weight and breed on pork quality. J. Anim. Sci. 91:1480–1492. doi: https://doi.org/10.2527/jas.2012-5200 23296818

[CIT0068] Van Der Heide, E. M. M., D. A. L. Lourenco, C. Y. Chen, W. O. Herring, R. L. Sapp, D. W. Moser, S. Tsuruta, Y. Masuda, B. J. Ducro, and I. Misztal. 2016. Sexual dimorphism in livestock species selected for economically important traits. J. Anim. Sci. 94:3684–3692. doi: https://doi.org/10.2527/jas.2016-0393 27898906

[CIT0069] Wagner-Golbs, A., S. Neuber, B. Kamlage, N. Christiansen, B. Bethan, U. Rennefahrt, P. Schatz, and L. Lind. 2019. Effects of Long-Term Storage at -80 °C on the Human Plasma Metabolome. Metabolites. 9:99. doi: https://doi.org/10.3390/metabo9050099 31108909 PMC6572224

[CIT0070] Wang, H., P. Xia, Z. Lu, Y. Su, and W. Zhu. 2021. Metabolome-microbiome responses of growing pigs induced by time-restricted feeding. Front. Vet. Sci. 8:681202. doi: https://doi.org/10.3389/fvets.2021.681202 34239912 PMC8258120

[CIT0071] Zamaratskaia, G., and E. J. Squires. 2009. Biochemical, nutritional and genetic effects on boar taint in entire male pigs. Animal. 3:1508–1521. doi: https://doi.org/10.1017/S1751731108003674 22444984

[CIT0072] Zhang, B., S. Hu, E. Baskin, A. Patt, J. K. Siddiqui, and E. A. Mathé. 2018. RaMP: a comprehensive relational database of metabolomics pathways for pathway enrichment analysis of genes and metabolites. Metabolites. 8:16. doi: https://doi.org/10.3390/metabo8010016 29470400 PMC5876005

