# Supplementary material for: Description of metabolic differences between castrated males and intact gilts obtained from high-throughput metabolomics of porcine plasma
Source: J Anim Sci. 2025 Mar 23;103:skaf178. doi: 10.1093/jas/skaf178 (PMC12202311; doi:10.1093/jas/skaf178)
Supplement: skaf178_suppl_Supplementary_Tables_S1-S3_Figure_S1 [file skaf178_suppl_supplementary_tables_s1-s3_figure_s1.docx]

**Supplementary material for:**

**Description of metabolic differences between castrated males and intact gilts obtained from high-throughput metabolomics of porcine plasma**

Samuele Bovo*^,1^, Matteo Bolner*^,1^, Giuseppina Schiavo*, Giuliano Galimberti^†^, Francesca Bertolini*, Stefania Dall’Olio*, Anisa Ribani*, Paolo Zambonelli*, Maurizio Gallo^‡^, Luca Fontanesi*^,2^

^*^Animal and Food Genomics Group, Division of Animal Sciences, Department of Agricultural and Food Sciences, University of Bologna, 40127, Bologna, Italy.

^†^Department of Statistical Sciences “Paolo Fortunati”, University of Bologna, 40126, Bologna, Italy.

^‡^Associazione Nazionale Allevatori Suini, 00198, Roma, Italy.

^1^These authors contributed equally to this work.

^2^Corresponding author: Luca Fontanesi: [luca.fontanesi@unibo.it](mailto:luca.fontanesi@unibo.it)

**Supplementary text S1.** Metabolon's experimental procedures

*Data Quality: Instrument and Process Variability –* Instrument variability was determined by calculating the median relative standard deviation (RSD) for the internal standards that were added to each sample prior to injection into the mass spectrometers. Overall process variability was determined by calculating the median RSD for all endogenous metabolites (i.e., non-instrument standards) present in 100% of the Client Matrix samples, which are technical replicates of pooled client samples. Values for instrument and process variability meet Metabolon’s acceptance criteria as shown in the Supplementary Text – Table S1 below.

**Supplementary Text – Table S1.** Metabolon’s acceptance criteria

| **Quality Control Sample** | **Median RSD**  **EDTA-K3 Plasma** |
| --- | --- |
| Internal Standards | 7% |
| Endogenous Biochemicals | 10% |

*Sample Accessioning –* Following receipt, samples were inventoried and immediately stored at -80°C. Each sample received was accessed into the Metabolon LIMS system and was assigned by the LIMS a unique identifier that was associated with the original source identifier only. This identifier was used to track all sample handling, tasks, results, etc. The samples (and all derived aliquots) were tracked by the LIMS system. All portions of any sample were automatically assigned their own unique identifiers by the LIMS when a new task was created; the relationship of these samples was also tracked. All samples were maintained at -80°C until processed.

*Sample Preparation –* Samples were prepared using the automated MicroLab STAR® system from Hamilton Company. Several recovery standards were added prior to the first step in the extraction process for QC purposes. To remove protein, dissociate small molecules bound to protein or trapped in the precipitated protein matrix, and to recover chemically diverse metabolites, proteins were precipitated with methanol under vigorous shaking for 2 min (Glen Mills GenoGrinder 2000) followed by centrifugation. The resulting extract was divided into multiple fractions: two for analysis by two separate reverse phase (RP)/UPLC-MS/MS methods with positive ion mode electrospray ionization (ESI), one for analysis by RP/UPLC-MS/MS with negative ion mode ESI, one for analysis by HILIC/UPLC-MS/MS with negative ion mode ESI, while the remaining fractions were reserved for backup. Samples were placed briefly on a TurboVap® (Zymark) to remove the organic solvent. The sample extracts were stored overnight under nitrogen before preparation for analysis.

*QA/QC –* Several types of controls were analyzed in concert with the experimental samples: a pooled matrix sample generated by taking a small volume of each experimental sample (or alternatively, use of a pool of well-characterized human plasma) served as a technical replicate throughout the data set; extracted water samples served as process blanks; and a cocktail of QC standards that were carefully chosen not to interfere with the measurement of endogenous compounds were spiked into every analyzed sample, allowed instrument performance monitoring and aided chromatographic alignment. Supplementary Text – Table S2 and S3 describe these QC samples and standards.

**Supplementary Text – Table S2.** Description of Metabolon QC Samples

| **Type** | **Description** | **Purpose** |
| --- | --- | --- |
| MTRX | Large pool of human plasma maintained by Metabolon that has been characterized extensively. | Assure that all aspects of the Metabolon process are operating within specifications. |
| CMTRX | Pool created by taking a small aliquot from every customer sample. | Assess the effect of a non-plasma matrix on the Metabolon process and distinguish biological variability from process variability. |
| PRCS | Aliquot of ultra-pure water | Process Blank used to assess the contribution to compound signals from the process. |

**Supplementary Text – Table S3**. Metabolon QC Standards.

| **Type** | **Description** | **Purpose** |
| --- | --- | --- |
| RS | Recovery Standard | Assess variability and verify performance of extraction and instrumentation. |
| IS | Internal Standard | Assess variability and performance of instrument. |

Instrument variability was determined by calculating the median relative standard deviation (RSD) for the standards that were added to each sample prior to injection into the mass spectrometers. Overall process variability was determined by calculating the median RSD for all endogenous metabolites (*i.e.,* non-instrument standards) present in 100% of the pooled matrix samples. Experimental samples were randomized across the platform run with QC samples spaced evenly among the injections, as outlined in the Supplementary Text – Figure S1 below.

**Supplementary Text – Figure S1.** Preparation of client-specific technical replicates. A small aliquot of each client sample (colored cylinders) is pooled to create a CMTRX technical replicate sample (multi-colored cylinder), which is then injected periodically throughout the platform run. Variability among consistently detected biochemicals can be used to calculate an estimate of overall process and platform variability.

*Ultrahigh Performance Liquid Chromatography-Tandem Mass Spectroscopy (UPLC-MS/MS) –* All methods utilized a Waters ACQUITY ultra-performance liquid chromatography (UPLC) and a Thermo Scientific Q-Exactive high resolution/accurate mass spectrometer interfaced with a heated electrospray ionization (HESI-II) source and Orbitrap mass analyzer operated at 35,000 mass resolution (Ford et al., 2020). The dried sample extract were then reconstituted in solvents compatible to each of the four methods. Each reconstitution solvent contained a series of standards at fixed concentrations to ensure injection and chromatographic consistency. One aliquot was analyzed using acidic positive ion conditions, chromatographically optimized for more hydrophilic compounds (PosEarly). In this method, the extract was gradient eluted from a C18 column (Waters UPLC BEH C18-2.1x100 mm, 1.7 µm) using water and methanol, containing 0.05% perfluoropentanoic acid (PFPA) and 0.1% formic acid (FA). Another aliquot was also analyzed using acidic positive ion conditions; however it was chromatographically optimized for more hydrophobic compounds (PosLate). In this method, the extract was gradient eluted from the same aforementioned C18 column using methanol, acetonitrile, water, 0.05% PFPA and 0.01% FA and was operated at an overall higher organic content. Another aliquot was analyzed using basic negative ion optimized conditions using a separate dedicated C18 column (Neg). The basic extracts were gradient eluted from the column using methanol and water, however with 6.5mM Ammonium Bicarbonate at pH 8. The fourth aliquot was analyzed via negative ionization following elution from a HILIC column (Waters UPLC BEH Amide 2.1x150 mm, 1.7 µm) using a gradient consisting of water and acetonitrile with 10mM Ammonium Formate, pH 10.8 (HILIC). The MS analysis alternated between MS and data-dependent MS^n^ scans using dynamic exclusion. The scan range varied slightly between methods but covered 70-1000 m/z. Raw data files are archived and extracted as described below.

*Bioinformatics –* The informatics system consisted of four major components, the Laboratory Information Management System (LIMS), the data extraction and peak-identification software, data processing tools for QC and compound identification, and a collection of information interpretation and visualization tools for use by data analysts. The hardware and software foundations for these informatics components were the LAN backbone, and a database server running Oracle 10.2.0.1 Enterprise Edition.

*LIMS –* The purpose of the Metabolon LIMS system was to enable fully auditable laboratory automation through a secure, easy to use, and highly specialized system. The scope of the Metabolon LIMS system encompasses sample accessioning, sample preparation and instrumental analysis and reporting and advanced data analysis. All of the subsequent software systems are grounded in the LIMS data structures. It has been modified to leverage and interface with the in-house information extraction and data visualization systems, as well as third party instrumentation and data analysis software.

*Data Extraction and Compound Identification –* Raw data was extracted, peak-identified and QC processed using a combination of Metabolon developed software services (applications). Each of these services perform a specific task independently, and they communicate/coordinate with each other using industry-standard protocols. Compounds were identified by comparison to library entries of purified standards or recurrent unknown entities. Metabolon maintains a library based on authenticated standards that contain the retention time/index (RI), mass to charge ratio (*m/z)*, and fragmentation data on all molecules present in the library. Furthermore, biochemical identifications are based on three criteria: retention index within a narrow RI window of the proposed identification, accurate mass match to the library +/- 10 ppm, and the MS/MS forward and reverse scores between the experimental data and authentic standards. The MS/MS scores are based on a comparison of the ions present in the experimental spectrum to the ions present in the library spectrum. While there may be similarities between molecules based on one of these factors, the use of all three data points is utilized to distinguish and differentiate biochemicals. More than 5,400 commercially available purified or in-house synthesized standard compounds have been acquired and analyzed on all platforms for determination of their analytical characteristics. An additional 7000 mass spectral entries have been created for structurally unnamed biochemicals, which have been identified by virtue of their recurrent nature (both chromatographic and mass spectral). These compounds have the potential to be identified by future acquisition of a matching purified standard or by classical structural analysis. Metabolon continuously adds biologically relevant compounds to its chemical library to further enhance its level of Tier 1 metabolite identifications.

*Compound Quality Control –* A variety of curation procedures were carried out to ensure that a high-quality data set was made available for statistical analysis and data interpretation. The QC and curation processes were designed to ensure accurate and consistent identification of true chemical entities, and to remove or correct those representing system artifacts, mis-assignments, mis-integration and background noise. Metabolon data analysts use proprietary visualization and interpretation software to confirm the consistency of peak identification and integration among the various samples.

*Metabolite Quantification and Data Normalization –* Peaks were quantified using area-under-the-curve. For studies spanning multiple days, a data normalization step was performed to correct variation resulting from instrument inter-day tuning differences. Essentially, each compound was corrected in run-day blocks by registering the medians to equal one (1.00) and normalizing each data point proportionately (termed the “block correction”, Supplementary Text – Figure S2). For studies that did not require more than one day of analysis, no normalization is necessary, other than for purposes of data visualization. In certain instances, biochemical data may have been normalized as an additional factor (*e.g.,* cell counts, total protein as determined by Bradford assay, osmolality, etc.) to account for differences in metabolite levels due to differences in the amount of material present in each sample.

**Supplementary Text – Figure S2**. Visualization of data normalization steps for a multiday platform run.

**References**

Ford L, Kennedy AD, Goodman KD, Pappan KL, Evans AM, Miller LAD, Wulff JE, Wiggs BR, Lennon JJ, Elsea S, Toal DR. Precision of a Clinical Metabolomics Profiling Platform for Use in the Identification of Inborn Errors of Metabolism. J Appl Lab Med. 2020 Mar 1;5(2):342-356. doi: 10.1093/jalm/jfz026. PMID: 32445384.

**Table S1.** Metabolites and their metabolisms evaluated in the study.

| **Super-pathway** | **Sub-pathway** | **Metabolites (n.)** | |
| --- | --- | --- | --- |
|  |  | **Raw dataset** | **Final dataset** |
| Unknown (Unnamed metabolites) | Unknown (Unnamed metabolites) | 69 | 64 |
| Amino Acid | Leucine, Isoleucine and Valine Metabolism | 28 | 26 |
| Lipid | Sphingomyelins | 25 | 25 |
| Lipid | Lysophospholipid | 23 | 23 |
| Lipid | Fatty Acid, Dicarboxylate | 19 | 19 |
| Lipid | Phosphatidylcholine (PC) | 19 | 16 |
| Lipid | Diacylglycerol | 18 | 11 |
| Amino Acid | Methionine, Cysteine, SAM and Taurine Metabolism | 18 | 16 |
| Amino Acid | Tryptophan Metabolism | 18 | 18 |
| Amino Acid | Urea cycle; Arginine and Proline Metabolism | 17 | 16 |
| Amino Acid | Histidine Metabolism | 17 | 15 |
| Lipid | Fatty Acid, Monohydroxy | 16 | 11 |
| Amino Acid | Lysine Metabolism | 15 | 15 |
| Lipid | Long Chain Polyunsaturated Fatty Acid (n3 and n6) | 14 | 12 |
| Lipid | Secondary Bile Acid Metabolism | 13 | 11 |
| Peptide | Gamma-glutamyl Amino Acid | 13 | 13 |
| Nucleotide | Pyrimidine Metabolism, Uracil containing | 11 | 11 |
| Lipid | Plasmalogen | 11 | 11 |
| Amino Acid | Tyrosine Metabolism | 11 | 11 |
| Amino Acid | Glycine, Serine and Threonine Metabolism | 10 | 10 |
| Lipid | Phosphatidylethanolamine (PE) | 9 | 9 |
| Nucleotide | Purine Metabolism, (Hypo)Xanthine/Inosine containing | 8 | 8 |
| Nucleotide | Pyrimidine Metabolism, Cytidine containing | 8 | 7 |
| Amino Acid | Glutamate Metabolism | 8 | 8 |
| Lipid | Long Chain Saturated Fatty Acid | 7 | 6 |
| Energy | TCA Cycle | 7 | 7 |
| Amino Acid | Alanine and Aspartate Metabolism | 7 | 7 |
| Lipid | Medium Chain Fatty Acid | 7 | 5 |
| Lipid | Phospholipid Metabolism | 7 | 7 |
| Partially Characterized Molecules | Partially Characterized Molecules | 7 | 5 |
| Nucleotide | Purine Metabolism, Adenine containing | 7 | 6 |
| Cofactors and Vitamins | Ascorbate and Aldarate Metabolism | 6 | 5 |
| Amino Acid | Glutathione Metabolism | 6 | 6 |
| Carbohydrate | Glycolysis, Gluconeogenesis, and Pyruvate Metabolism | 6 | 6 |
| Amino Acid | Polyamine Metabolism | 6 | 5 |
| Lipid | Long Chain Monounsaturated Fatty Acid | 6 | 6 |
| Peptide | Dipeptide | 6 | 5 |
| Lipid | Fatty Acid Metabolism (Acyl Glycine) | 6 | 4 |
| Cofactors and Vitamins | Nicotinate and Nicotinamide Metabolism | 6 | 5 |
| Amino Acid | Lactoyl Amino Acid | 5 | 4 |
| Amino Acid | Phenylalanine Metabolism | 5 | 5 |
| Carbohydrate | Pentose Metabolism | 5 | 5 |
| Lipid | Phosphatidylinositol (PI) | 5 | 5 |
| Lipid | Fatty Acid Metabolism (Acyl Choline) | 5 | 0 |
| Lipid | Ceramides | 5 | 3 |
| Lipid | Dihydrosphingomyelins | 5 | 5 |
| Lipid | Sterol | 5 | 5 |
| Nucleotide | Purine Metabolism, Guanine containing | 5 | 5 |
| Carbohydrate | Aminosugar Metabolism | 5 | 5 |
| Amino Acid | Creatine Metabolism | 4 | 4 |
| Carbohydrate | Fructose, Mannose and Galactose Metabolism | 4 | 4 |
| Lipid | Lysoplasmalogen | 4 | 4 |
| Lipid | Endocannabinoid | 4 | 4 |
| Peptide | Acetylated Peptides | 4 | 4 |
| Lipid | Hexosylceramides (HCER) | 4 | 4 |
| Lipid | Fatty Acid, Dihydroxy | 4 | 4 |
| Lipid | Fatty Acid Metabolism (Acyl Carnitine, Long Chain Saturated) | 4 | 3 |
| Lipid | Fatty Acid Metabolism (also BCAA Metabolism) | 3 | 3 |
| Lipid | Primary Bile Acid Metabolism | 3 | 3 |
| Lipid | Corticosteroids | 3 | 3 |
| Lipid | Fatty Acid Metabolism (Acyl Carnitine, Monounsaturated) | 3 | 3 |
| Lipid | Fatty Acid Metabolism (Acyl Carnitine, Medium Chain) | 3 | 2 |
| Cofactors and Vitamins | Hemoglobin and Porphyrin Metabolism | 3 | 1 |
| Cofactors and Vitamins | Tocopherol Metabolism | 3 | 2 |
| Lipid | Fatty Acid, Amino | 3 | 3 |
| Lipid | Glycerolipid Metabolism | 3 | 3 |
| Lipid | Fatty Acid Metabolism (Acyl Carnitine, Dicarboxylate) | 3 | 2 |
| Peptide | Dipeptide Derivative | 2 | 2 |
| Nucleotide | Pyrimidine Metabolism, Thymine containing | 2 | 2 |
| Lipid | Sphingosines | 2 | 2 |
| Nucleotide | Pyrimidine Metabolism, Orotate containing | 2 | 1 |
| Lipid | Sphingolipid Synthesis | 2 | 2 |
| Lipid | Mevalonate Metabolism | 2 | 2 |
| Lipid | Fatty Acid, Branched | 2 | 0 |
| Lipid | Inositol Metabolism | 2 | 2 |
| Cofactors and Vitamins | Pantothenate and CoA Metabolism | 2 | 2 |
| Amino Acid | Guanidino and Acetamido Metabolism | 2 | 2 |
| Lipid | Carnitine Metabolism | 2 | 2 |
| Lipid | Dihydroceramides | 2 | 2 |
| Cofactors and Vitamins | Riboflavin Metabolism | 1 | 1 |
| Cofactors and Vitamins | Pterin Metabolism | 1 | 1 |
| Carbohydrate | Advanced Glycation End-product | 1 | 1 |
| Carbohydrate | Disaccharides and Oligosaccharides | 1 | 0 |
| Lipid | Ceramide PEs | 1 | 1 |
| Energy | Oxidative Phosphorylation | 1 | 1 |
| Cofactors and Vitamins | Vitamin A Metabolism | 1 | 1 |
| Cofactors and Vitamins | Vitamin B6 Metabolism | 1 | 1 |
| Lipid | Fatty Acid Metabolism (Acyl Carnitine, Polyunsaturated) | 1 | 1 |
| Lipid | Fatty Acid Metabolism (Acyl Carnitine, Hydroxy) | 1 | 0 |
| Lipid | Glycosphingolipid Sulfates | 1 | 1 |
| Lipid | Fatty Acid Metabolism (Acyl Carnitine, Short Chain) | 1 | 1 |
| Lipid | Ketone Bodies | 1 | 1 |
| Lipid | Lactosylceramides (LCER) | 1 | 1 |
| Lipid | Monoacylglycerol | 1 | 1 |
| Lipid | Phosphatidylserine (PS) | 1 | 1 |
| Lipid | Short Chain Fatty Acid | 1 | 1 |
| Lipid | Progestin Steroids | 1 | 0 |
| Nucleotide | Purine and Pyrimidine Metabolism | 1 | 1 |
| Peptide | Polypeptide | 1 | 0 |

**Table S2.** Overlap in terms of metabolites between the Biocrates and Metabolon platforms.

| **Biocrates name** | **Metabolon name** | **Metabolon**  **Super-pathway** | **Metabolon Sub-pathway** | ***r*^1^** |
| --- | --- | --- | --- | --- |
| Ala | alanine | Amino Acid | Alanine and Aspartate Metabolism | 0.78 |
| Asn | asparagine | Amino Acid | Alanine and Aspartate Metabolism | 0.61 |
| Creatinine | creatinine | Amino Acid | Creatine Metabolism | 0.74 |
| Glu | glutamate | Amino Acid | Glutamate Metabolism | 0.74 |
| Gln | glutamine | Amino Acid | Glutamate Metabolism | 0.65 |
| Gly | glycine | Amino Acid | Glycine, Serine and Threonine Metabolism | 0.74 |
| Sarcosine | sarcosine | Amino Acid | Glycine, Serine and Threonine Metabolism | 0.43 |
| Ser | serine | Amino Acid | Glycine, Serine and Threonine Metabolism | 0.63 |
| Thr | threonine | Amino Acid | Glycine, Serine and Threonine Metabolism | 0.80 |
| His | histidine | Amino Acid | Histidine Metabolism | 0.44 |
| Ile | isoleucine | Amino Acid | Leucine, Isoleucine and Valine Metabolism | 0.74 |
| Leu | leucine | Amino Acid | Leucine, Isoleucine and Valine Metabolism | 0.68 |
| Val | valine | Amino Acid | Leucine, Isoleucine and Valine Metabolism | 0.54 |
| alpha-AAA | 2-aminoadipate | Amino Acid | Lysine Metabolism | 0.66 |
| Lys | lysine | Amino Acid | Lysine Metabolism | 0.59 |
| Met | methionine | Amino Acid | Methionine, Cysteine, SAM and Taurine Metabolism | 0.71 |
| Met:SO | methionine sulfoxide | Amino Acid | Methionine, Cysteine, SAM and Taurine Metabolism | 0.57 |
| Taurine | taurine | Amino Acid | Methionine, Cysteine, SAM and Taurine Metabolism | 0.79 |
| Phe | phenylalanine | Amino Acid | Phenylalanine Metabolism | 0.66 |
| Kynurenine | kynurenine | Amino Acid | Tryptophan Metabolism | 0.87 |
| Serotonin | serotonin | Amino Acid | Tryptophan Metabolism | 0.73 |
| Trp | tryptophan | Amino Acid | Tryptophan Metabolism | 0.71 |
| Tyr | tyrosine | Amino Acid | Tyrosine Metabolism | 0.70 |
| Arg | arginine | Amino Acid | Urea cycle; Arginine and Proline Metabolism | 0.72 |
| Cit | citrulline | Amino Acid | Urea cycle; Arginine and Proline Metabolism | 0.78 |
| Orn | ornithine | Amino Acid | Urea cycle; Arginine and Proline Metabolism | 0.62 |
| Pro | proline | Amino Acid | Urea cycle; Arginine and Proline Metabolism | 0.76 |
| H1 | glucose | Carbohydrate | Glycolysis, Gluconeogenesis, and Pyruvate Metabolism | 0.74 |
| C0 | carnitine | Lipid | Carnitine Metabolism | 0.81 |
| C16 | palmitoylcarnitine (C16) | Lipid | Fatty Acid Metabolism (Acyl Carnitine, Long Chain Saturated) | 0.85 |
| C18 | stearoylcarnitine (C18) | Lipid | Fatty Acid Metabolism (Acyl Carnitine, Long Chain Saturated) | 0.84 |
| C18:1 | oleoylcarnitine (C18:1) | Lipid | Fatty Acid Metabolism (Acyl Carnitine, Monounsaturated) | 0.88 |
| C18:2 | linoleoylcarnitine (C18:2) | Lipid | Fatty Acid Metabolism (Acyl Carnitine, Polyunsaturated) | 0.76 |
| C2 | acetylcarnitine (C2) | Lipid | Fatty Acid Metabolism (Acyl Carnitine, Short Chain) | 0.72 |
| C3 | propionylcarnitine (C3) | Lipid | Fatty Acid Metabolism (also BCAA Metabolism) | 0.84 |
| lysoPC a C18:2 | 1-linoleoyl-GPC (18:2) | Lipid | Lysophospholipid | 0.78 |
| lysoPC a C18:1 | 1-oleoyl-GPC (18:1) | Lipid | Lysophospholipid | 0.67 |
| lysoPC a C16:1 | 1-palmitoleoyl-GPC (16:1) | Lipid | Lysophospholipid | 0.58 |
| lysoPC a C16:0 | 1-palmitoyl-GPC (16:0) | Lipid | Lysophospholipid | 0.68 |
| lysoPC a C18:0 | 1-stearoyl-GPC (18:0) | Lipid | Lysophospholipid | 0.66 |
| PC aa C34:4 | 1-myristoyl-2-arachidonoyl-GPC (14:0/20:4) | Lipid | Phosphatidylcholine (PC) | 0.70 |
| PC aa C36:5 | 1-palmitoyl-2-arachidonoyl-GPC (16:0/20:4n6) | Lipid | Phosphatidylcholine (PC) | 0.68 |
| PC aa C38:6 | 1-palmitoyl-2-docosahexaenoyl-GPC (16:0/22:6) | Lipid | Phosphatidylcholine (PC) | 0.79 |
| PC aa C34:2 | 1-palmitoyl-2-linoleoyl-GPC (16:0/18:2) | Lipid | Phosphatidylcholine (PC) | 0.82 |
| PC aa C34:1 | 1-palmitoyl-2-oleoyl-GPC (16:0/18:1) | Lipid | Phosphatidylcholine (PC) | 0.77 |
| PC aa C32:1 | 1-palmitoyl-2-palmitoleoyl-GPC (16:0/16:1) | Lipid | Phosphatidylcholine (PC) | 0.77 |
| PC aa C38:4 | 1-stearoyl-2-arachidonoyl-GPC (18:0/20:4) | Lipid | Phosphatidylcholine (PC) | 0.72 |
| PC aa C40:6 | 1-stearoyl-2-docosahexaenoyl-GPC (18:0/22:6) | Lipid | Phosphatidylcholine (PC) | 0.88 |
| PC aa C36:2 | 1-stearoyl-2-linoleoyl-GPC (18:0/18:2) | Lipid | Phosphatidylcholine (PC) | 0.75 |
| PC aa C36:1 | 1-stearoyl-2-oleoyl-GPC (18:0/18:1) | Lipid | Phosphatidylcholine (PC) | 0.81 |
| SM C16:0 | palmitoyl sphingomyelin (d18:1/16:0) | Lipid | Sphingomyelins | 0.56 |
| SM C18:1 | sphingomyelin (d18:1/18:1, d18:2/18:0) | Lipid | Sphingomyelins | 0.75 |
| SM C16:1 | sphingomyelin (d18:2/16:0, d18:1/16:1) | Lipid | Sphingomyelins | 0.67 |
| SM C18:0 | stearoyl sphingomyelin (d18:1/18:0) | Lipid | Sphingomyelins | 0.70 |

^1^ Pearson correlation coefficient (*r*) between absolute (Biocrates) and relative (Metabolon) metabolite quantification.

**Table S3.** Re-analysis of Biocrates panel: evaluation of the statistical pipelines.

|  | **sPLS-DA (novel pipeline)^2^** | | | **Boruta (novel pipeline)^3^** | | **sPLS-DA (old pipeline)^4^** | | |  |
| --- | --- | --- | --- | --- | --- | --- | --- | --- | --- |
| **Metabolite^1^** | **Sel** | **Conf.** | ***β-*conc** | **Sel** | **Conf.** | **Pst** | ***β*** | **Psg** | **AUC^5^** |
| C3 | 5 | 49 | 1 | 5 | 50* | 0.020 | -0.135 | 0.055 | 0.619 |
| PC ae C36:5 | 5 | 50* | 1 | 5 | 50* | 0.006 | 0.200 | 0.019 | 0.6022 |
| PC aa C40:6 | 5 | 47 | 1 | 5 | 50* | 0.013 | 0.077 | 0.072 | 0.597 |
| PC ae C36:1 | 5 | 18 | 1 | 5 | 43 | 0.007 | -0.116 | 0.018 | 0.596 |
| Serotonin | 5 | 50* | 1 | 5 | 50* | 0.014 | -0.206 | 0.065 | 0.593 |
| Kynurenine | 5 | 50* | 1 | 5 | 38 | 0.011 | -0.142 | 0.068 | 0.5909 |
| Met:SO | 5 | 50* | 1 | 0 | 12 | 0.019 | -0.135 | 0.070 | 0.5889 |
| lysoPC a C17:0 | 5 | 49 | 1 | 0 | 12 | 0.022 | -0.133 | 0.054 | 0.5867 |
| SM (OH) C16:1 | 5 | 50* | 1 | 2 | 12 | 0.010 | -0.184 | 0.018 | 0.5862 |
| PC ae C44:6 | 5 | 50* | 1 | 4 | 24 | 0.013 | 0.121 | 0.061 | 0.5783 |
| Taurine | 5 | 50* | 1 | 0 | 14 | 0.019 | -0.159 | 0.068 | 0.5782 |
| SM C18:1 | 5 | 50* | 1 | 2 | 13 | 0.014 | 0.130 | 0.048 | 0.5755 |
| PC aa C32:2 | 5 | 50* | 1 | 0 | 0 | 0.010 | -0.108 | 0.032 | 0.5722 |
| PC ae C36:2 | 5 | 30 | 1 | 0 | 0 | 0.003 | -0.059 | 0.033 | 0.5701 |
| His | 5 | 50* | 1 | 0 | 13 | 0.011 | 0.183 | 0.036 | 0.5687 |
| Leu | 5 | 49 | 1 | 0 | 2 | 0.014 | -0.092 | 0.062 | 0.5683 |
| PC aa C38:6 | 5 | 47 | 1 | 0 | 7 | 0.010 | 0.094 | 0.041 | 0.5652 |
| C2 | 3 | 19 | 1 | 0 | 0 | 0.009 | 0.012 | 0.169 | 0.5649 |
| PC aa C34:4 | 5 | 47 | 1 | 1 | 10 | 0.024 | -0.100 | 0.031 | 0.5631 |
| PC ae C38:1 | 5 | 47 | 1 | 2 | 23 | 0.009 | -0.114 | 0.033 | 0.5626 |
| PC ae C34:2 | 5 | 49 | 1 | 0 | 6 | 0.011 | -0.163 | 0.009 | 0.5625 |
| C16-OH | 5 | 48 | 1 | 0 | 0 | 0.017 | -0.118 | 0.099 | 0.562 |
| Ser | 5 | 50* | 1 | 3 | 20 | 0.016 | 0.192 | 0.052 | 0.5594 |
| Ile | 4 | 13 | 0 | 0 | 0 | 0.014 | -0.074 | 0.081 | 0.5582 |
| PC aa C38:0 | 5 | 38 | 1 | 4 | 26 | 0.015 | 0.013 | 0.118 | 0.5565 |
| C0 | 4 | 11 | 1 | 0 | 2 | 0.020 | -0.038 | 0.140 | 0.5546 |
| C16:1 | 4 | 18 | 0 | 0 | 0 | 0.019 | -0.056 | 0.140 | 0.5539 |
| H1 | 3 | 26 | 1 | 0 | 0 | 0.015 | -0.040 | 0.125 | 0.5536 |
| PC ae C44:5 | 4 | 12 | 1 | 0 | 0 | 0.010 | 0.049 | 0.116 | 0.5516 |
| lysoPC a C18:0 | 5 | 17 | 0 | 0 | 0 | 0.012 | 0.033 | 0.110 | 0.55 |
| C14 | 5 | 23 | 1 | 0 | 0 | 0.010 | -0.105 | 0.082 | 0.55 |
| C16 | 5 | 49 | 1 | 0 | 0 | 0.017 | -0.156 | 0.035 | 0.5497 |
| PC aa C42:0 | 5 | 47 | 1 | 0 | 5 | 0.010 | 0.053 | 0.063 | 0.5496 |
| SM C16:1 | 4 | 17 | 0 | 0 | 0 | 0.012 | 0.051 | 0.079 | 0.5488 |
| PC aa C32:1 | 2 | 12 | 1 | 0 | 0 | 0.017 | -0.015 | 0.119 | 0.5488 |
| PC ae C34:1 | 5 | 15 | 0 | 0 | 2 | 0.003 | -0.066 | 0.033 | 0.5475 |
| PC ae C40:6 | 5 | 35 | 1 | 0 | 0 | 0.015 | -0.005 | 0.139 | 0.5462 |
| PC ae C38:6 | 5 | 30 | 1 | 0 | 0 | 0.007 | 0.018 | 0.099 | 0.544 |
| SM C24:1 | 4 | 37 | 1 | 0 | 0 | 0.014 | 0.093 | 0.084 | 0.5439 |
| PC ae C44:4 | 5 | 47 | 1 | 0 | 0 | 0.012 | 0.047 | 0.110 | 0.5431 |
| Spermidine | 4 | 39 | 1 | 0 | 0 | 0.017 | -0.128 | 0.086 | 0.5427 |
| PC aa C34:3 | 2 | 17 | 1 | 0 | 0 | 0.027 | -0.042 | 0.082 | 0.5421 |
| Val | 2 | 18 | 1 | 0 | 0 | 0.026 | -0.036 | 0.129 | 0.5419 |
| C18:1 | 3 | 28 | 1 | 0 | 0 | 0.011 | -0.122 | 0.045 | 0.5413 |
| lysoPC a C20:4 | 5 | 47 | 1 | 1 | 6 | 0.009 | 0.071 | 0.089 | 0.5402 |
| C14:1 | 3 | 14 | 1 | 0 | 0 | 0.012 | -0.060 | 0.120 | 0.5397 |
| SM (OH )C22:1 | 3 | 27 | 1 | 0 | 0 | 0.011 | -0.133 | 0.040 | 0.5389 |
| C18:1-OH | 3 | 10 | 0 | 0 | 0 | 0.011 | -0.070 | 0.122 | 0.5385 |
| PC ae C36:3 | 4 | 38 | 1 | 0 | 0 | 0.008 | -0.088 | 0.027 | 0.5384 |
| PC ae C42:0 | 3 | 28 | 1 | 0 | 0 | 0.010 | 0.033 | 0.133 | 0.5383 |
| Cit | 5 | 34 | 0 | 0 | 0 | 0.023 | -0.067 | 0.125 | 0.5375 |
| Ala | 3 | 16 | 0 | 0 | 2 | 0.014 | 0.119 | 0.061 | 0.5369 |
| PC ae C38:2 | 2 | 11 | 1 | 0 | 0 | 0.011 | -0.026 | 0.089 | 0.5367 |
| Gly | 2 | 14 | 1 | 0 | 0 | 0.013 | 0.077 | 0.098 | 0.5367 |
| Thr | 5 | 47 | 1 | 0 | 0 | 0.021 | 0.157 | 0.050 | 0.5362 |
| lysoPC a C18:2 | 3 | 27 | 1 | 0 | 0 | 0.008 | 0.112 | 0.059 | 0.5354 |
| PC ae C40:1 | 5 | 25 | 0 | 0 | 0 | 0.017 | 0.005 | 0.154 | 0.5351 |
| PC aa C36:6 | 2 | 16 | 1 | 0 | 0 | 0.028 | -0.081 | 0.071 | 0.533 |
| PC aa C36:2 | 3 | 27 | 1 | 0 | 0 | 0.009 | 0.111 | 0.021 | 0.5321 |
| PC ae C38:5 | 3 | 20 | 1 | 0 | 0 | 0.011 | 0.005 | 0.135 | 0.5318 |
| PC aa C30:0 | 3 | 28 | 1 | 0 | 0 | 0.009 | 0.156 | 0.020 | 0.5317 |
| Sarcosine | 5 | 27 | 1 | 0 | 0 | 0.016 | -0.064 | 0.138 | 0.5315 |
| PC aa C38:3 | 2 | 16 | 1 | 0 | 0 | 0.004 | 0.084 | 0.044 | 0.5305 |
| PC aa C34:1 | 2 | 13 | 1 | 0 | 0 | 0.011 | 0.000 | 1.000 | 0.5304 |
| PC aa C40:2 | 2 | 16 | 1 | 0 | 0 | 0.015 | -0.094 | 0.026 | 0.53 |
| PC aa C40:4 | 2 | 16 | 1 | 0 | 0 | 0.020 | 0.000 | 1.000 | 0.5293 |
| PC ae C38:3 | 2 | 12 | 1 | 0 | 0 | 0.015 | -0.014 | 0.104 | 0.5287 |
| Spermine | 2 | 9 | 1 | 0 | 0 | 0.049 | 0.000 | 1.000 | 0.528 |
| PC aa C36:5 | 2 | 11 | 1 | 0 | 0 | 0.073 | 0.000 | 1.000 | 0.5277 |
| Carnosine | 2 | 6 | 1 | 0 | 0 | 0.030 | 0.021 | 0.150 | 0.5277 |
| PC ae C40:2 | 2 | 16 | 1 | 0 | 0 | 0.017 | -0.090 | 0.026 | 0.5276 |
| PC aa C38:4 | 4 | 18 | 0 | 0 | 0 | 0.006 | 0.008 | 0.122 | 0.5274 |
| alpha:AAA | 3 | 11 | 0 | 0 | 0 | 0.014 | -0.026 | 0.145 | 0.5274 |
| lysoPC:a:C16:1 | 0 | 0 | 0 | 0 | 0 | 0.050 | 0.000 | 1.000 | 0.5273 |
| Phe | 2 | 15 | 1 | 0 | 0 | 0.020 | 0.000 | 1.000 | 0.5267 |
| PC ae C32:2 | 2 | 15 | 1 | 0 | 0 | 0.017 | 0.000 | 1.000 | 0.526 |
| SM (OH)C24:1 | 2 | 15 | 1 | 0 | 0 | 0.009 | 0.033 | 0.108 | 0.5258 |
| SM (OH) C14:1 | 2 | 5 | 1 | 0 | 0 | 0.027 | 0.000 | 1.000 | 0.5258 |
| PC ae C34:0 | 2 | 11 | 0 | 0 | 0 | 0.043 | 0.000 | 1.000 | 0.5254 |
| lysoPC a C20:3 | 2 | 16 | 1 | 0 | 0 | 0.016 | 0.128 | 0.059 | 0.5248 |
| SM C18:0 | 2 | 17 | 1 | 0 | 0 | 0.012 | 0.040 | 0.110 | 0.5247 |
| PC ae C42:2 | 5 | 40 | 1 | 0 | 0 | 0.006 | 0.022 | 0.108 | 0.5246 |
| PC ae C42:5 | 2 | 14 | 1 | 0 | 0 | 0.010 | -0.012 | 0.135 | 0.5239 |
| SM C24:0 | 2 | 16 | 1 | 0 | 0 | 0.012 | 0.052 | 0.059 | 0.5237 |
| Creatinine | 2 | 11 | 1 | 0 | 0 | 0.027 | 0.000 | 1.000 | 0.5233 |
| Trp | 2 | 16 | 1 | 0 | 0 | 0.013 | 0.095 | 0.080 | 0.5224 |
| Arg | 2 | 15 | 1 | 0 | 0 | 0.016 | 0.101 | 0.073 | 0.5214 |
| PC aa C36:3 | 2 | 6 | 1 | 0 | 0 | 0.056 | 0.000 | 1.000 | 0.5199 |
| Gln | 2 | 10 | 1 | 0 | 0 | 0.015 | 0.000 | 1.000 | 0.5198 |
| PC aa C38:5 | 2 | 14 | 1 | 0 | 0 | 0.007 | 0.000 | 1.000 | 0.5192 |
| PC aa C40:3 | 2 | 13 | 1 | 0 | 0 | 0.037 | 0.000 | 1.000 | 0.5182 |
| C18:2 | 2 | 7 | 1 | 0 | 0 | 0.057 | 0.000 | 1.000 | 0.5171 |
| C18 | 2 | 2 | 1 | 0 | 0 | 0.059 | 0.000 | 1.000 | 0.516 |
| PC ae C42:4 | 2 | 6 | 1 | 0 | 0 | 0.032 | 0.000 | 1.000 | 0.5155 |
| PC aa C42:2 | 2 | 14 | 1 | 0 | 0 | 0.007 | 0.039 | 0.079 | 0.5154 |
| PC ae C40:3 | 2 | 8 | 1 | 0 | 0 | 0.050 | 0.000 | 1.000 | 0.515 |
| SM (OH) C22:2 | 2 | 13 | 1 | 0 | 0 | 0.079 | 0.000 | 1.000 | 0.5147 |
| Putrescine | 2 | 17 | 1 | 0 | 0 | 0.027 | 0.093 | 0.081 | 0.5146 |
| Glu | 2 | 15 | 1 | 0 | 0 | 0.031 | 0.000 | 1.000 | 0.5146 |
| PC aa C36:0 | 2 | 12 | 1 | 0 | 0 | 0.006 | 0.000 | 1.000 | 0.5145 |
| lysoPC a C14:0 | 2 | 12 | 1 | 0 | 0 | 0.053 | 0.000 | 1.000 | 0.5134 |
| PC aa C34:2 | 1 | 8 | 1 | 0 | 0 | 0.031 | 0.000 | 1.000 | 0.5128 |
| PC aa C36:1 | 2 | 8 | 0 | 0 | 0 | 0.070 | 0.000 | 1.000 | 0.5121 |
| Tyr | 2 | 4 | 1 | 0 | 0 | 0.058 | 0.000 | 1.000 | 0.5113 |
| PC aa C28:1 | 2 | 12 | 1 | 0 | 0 | 0.105 | 0.000 | 1.000 | 0.5113 |
| PC aa C42:6 | 2 | 13 | 1 | 0 | 0 | 0.066 | 0.000 | 1.000 | 0.5098 |
| PC aa C40:5 | 0 | 0 | 0 | 0 | 0 | 0.038 | 0.000 | 1.000 | 0.5096 |
| PC ae C32:1 | 2 | 13 | 1 | 0 | 0 | 0.031 | 0.000 | 1.000 | 0.5095 |
| PC ae C34:3 | 2 | 14 | 1 | 0 | 0 | 0.055 | 0.000 | 1.000 | 0.5092 |
| PC ae C36:4 | 2 | 10 | 1 | 0 | 0 | 0.027 | 0.000 | 1.000 | 0.509 |
| PC ae C30:0 | 2 | 8 | 1 | 0 | 0 | 0.048 | 0.000 | 1.000 | 0.5087 |
| PC aa C32:0 | 2 | 7 | 0 | 0 | 0 | 0.044 | 0.000 | 1.000 | 0.5069 |
| SM:C16:0 | 2 | 13 | 1 | 0 | 0 | 0.039 | 0.000 | 1.000 | 0.5058 |
| PC aa C40:1 | 2 | 6 | 1 | 0 | 0 | 0.052 | 0.000 | 1.000 | 0.5054 |
| C12:DC | 2 | 8 | 1 | 0 | 0 | 0.051 | 0.000 | 1.000 | 0.505 |
| Asn | 1 | 4 | 1 | 0 | 0 | 0.109 | 0.000 | 1.000 | 0.5042 |
| Pro | 2 | 7 | 1 | 0 | 0 | 0.090 | 0.000 | 1.000 | 0.5017 |
| lysoPC a C18:1 | 2 | 10 | 1 | 0 | 0 | 0.040 | 0.000 | 1.000 | 0.5016 |
| PC aa C42:5 | 2 | 12 | 1 | 0 | 0 | 0.040 | 0.000 | 1.000 | 0.5011 |
| Met | 2 | 13 | 1 | 0 | 0 | 0.029 | 0.000 | 1.000 | 0.4994 |
| PC ae C40:5 | 1 | 2 | 1 | 0 | 0 | 0.048 | 0.000 | 1.000 | 0.4986 |
| PC ae C38:4 | 2 | 6 | 1 | 0 | 0 | 0.019 | 0.000 | 1.000 | 0.4971 |
| Orn | 2 | 9 | 0 | 0 | 0 | 0.090 | 0.000 | 1.000 | 0.497 |
| PC aa C36:4 | 2 | 16 | 1 | 0 | 0 | 0.008 | 0.098 | 0.031 | 0.4955 |
| PC ae C40:4 | 2 | 4 | 1 | 0 | 0 | 0.030 | 0.000 | 1.000 | 0.4938 |
| lysoPC a C16:0 | 2 | 5 | 1 | 0 | 0 | 0.019 | 0.000 | 1.000 | 0.4928 |
| PC ae C36:0 | 0 | 0 | 0 | 0 | 0 | 0.148 | 0.000 | 1.000 | 0.4918 |
| Lys | 2 | 18 | 1 | 0 | 0 | 0.021 | 0.121 | 0.059 | 0.4892 |
| PC aa C42:4 | 3 | 18 | 0 | 0 | 0 | 0.008 | 0.041 | 0.079 | 0.4807 |
| PC aa C42:1 | 2 | 10 | 1 | 0 | 0 | 0.007 | -0.015 | 0.121 | 0.4807 |
| PC ae C38:0 | 5 | 26 | 0 | 0 | 0 | 0.009 | 0.011 | 0.126 | 0.4713 |
| PC ae C42:3 | 5 | 37 | 1 | 0 | 0 | 0.012 | -0.001 | 0.145 | 0.4628 |

^1^ Metabolite information is provided in Bovo et al., 2015. Metabolites are ranked by the area under the curve (AUC) values;

^2^ Novel pipeline of selection relying on sparse partial least squares discriminant analysis (sPLS-DA). Sel: no. of runs (out of five) the metabolite is selected as discriminant; Sel: no. of runs (out of 50) the metabolite is selected and confirmed as discriminant across the external 10CV procedure; *β*-conc: 1 indicates the *β* coefficient of regression presenting the same direction across the 50 runs while 0 indicates the *β* coefficient of regression presenting the same direction across the 50 runs; the symbol star (*) indicates the metabolites of the core set;

^3^ Novel pipeline of selection relying on Boruta. Sel: no. of runs (out of five) the metabolite is selected as discriminant; Sel: no. of runs (out of 50) the metabolite is selected and confirmed as discriminant across the external 10CV procedure; the symbol star (*) indicates the metabolites of the core set;

^4^ Old pipeline of selection relying on sPLS-DA. Pst: *P* at the stability test; *β*: regression coefficient; Psg: *P* at the significance test. Details are given by Bovo et al., 2015.

^4^ AUC: Area under the curve of the receiver operating characteristic analysis.


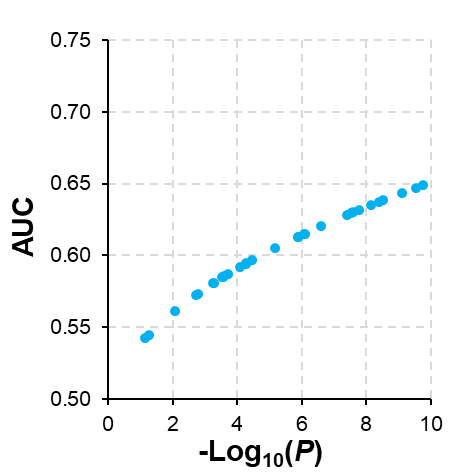


**Figure S1.** Relationship between area under the curve (AUC) and Mann-Whitney U-statistic (*P*) for the 40 sex-influenced metabolites.
